# Transcriptional profiling of ErbB signalling in mammary luminal epithelial cells - interplay of ErbB and IGF1 signalling through IGFBP3 regulation

**DOI:** 10.1186/1471-2407-10-490

**Published:** 2010-09-14

**Authors:** Jenny Worthington, Mariana Bertani, Hong-Lin Chan, Bertran Gerrits, John F Timms

**Affiliations:** 1 Cancer Proteomics Laboratory, EGA Institute for Women's Health, University College London, Cruciform Building, Gower Street, London, WC1E 6BT, UK; 2 Ludwig Institute for Cancer Research, Cruciform Building, Gower Street, London, WC1E 6BT, UK; 3 Institute of Bioinformatics and Structural Biology, National Tsing Hua University, Hsinchu, Taiwan

## Abstract

**Background:**

Members of the ErbB family of growth factor receptors are intricately linked with epithelial cell biology, development and tumourigenesis; however, the mechanisms involved in their downstream signalling are poorly understood. Indeed, it is unclear how signal specificity is achieved and the relative contribution each receptor has to specific gene expression.

**Methods:**

Gene expression profiling of a human mammary luminal epithelial cell model of ErbB2-overexpression was carried out using cDNA microarrays with a common RNA reference approach to examine long-term overlapping and differential responses to EGF and heregulin beta1 treatment in the context of ErbB2 overexpression. Altered gene expression was validated using quantitative real time PCR and/or immunoblotting. One gene of interest was targeted for further characterisation, where the effects of siRNA-mediated silencing on IGF1-dependent signalling and cellular phenotype were examined and compared to the effects of loss of ErbB2 expression.

**Results:**

775 genes were differentially expressed and clustered in terms of their growth factor responsiveness. As well as identifying uncharacterized genes as novel targets of ErbB2-dependent signalling, ErbB2 overexpression augmented the induction of multiple genes involved in proliferation (e.g. MYC, MAP2K1, MAP2K3), autocrine growth factor signalling (VEGF, PDGF) and adhesion/cytoskeletal regulation (ZYX, THBS1, VCL, CNN3, ITGA2, ITGA3, NEDD9, TAGLN), linking them to the hyper-poliferative and altered adhesive phenotype of the ErbB2-overexpressing cells. We also report ErbB2-dependent down-regulation of multiple interferon-stimulated genes that may permit ErbB2-overexpressing cells to resist the anti-proliferative action of interferons. Finally, IGFBP3 was unique in its pattern of regulation and we further investigated a possible role for IGFBP3 down-regulation in ErbB2-dependent transformation through suppressed IGF1 signalling. We show that IGF1-dependent signalling and proliferation were enhanced in ErbB2-overexpressing cells, whilst loss of ErbB2 expression by siRNA silencing reduced IGF1 signalling. Furthermore, IGFBP3 knockdown resulted in basal ERK and Akt activation in luminal epithelial cells and increased invasiveness and anchorage-independent colony formation in SKBR3 cells.

**Conclusions:**

These data show IGFBP3 as a negative regulator of transformation and that its down-regulation enhances IGF1-dependent signalling. They also show that ErbB2 can up-regulate IGF1-dependent signalling, possibly via the regulated expression of IGFBP3.

## Background

The expression and activity of the ErbB/HER family of receptor tyrosine kinases is frequently deregulated in human cancers. To date, four members of this family have been described: EGFR, ErbB2 (HER2), ErbB3 (HER3) and ErbB4 (HER4). Signalling through the ErbB family is initiated by ligand-induced receptor homo- or heterodimerzation leading to stimulation of the receptors' intrinsic tyrosine kinase activity and triggering of auto- and cross-phosphorylation of tyrosine residues creating docking sites for adaptor proteins and enzymes that initiate signal transduction events ultimately leading to changes in gene expression and altered cellular phenotype [1]. Numerous tumour, epithelial or stromal-derived growth factors (GFs) bind with different affinities and specificities to the different ErbB family members. These include: EGF, TGFα and amphiregulin (AREG), which bind specifically to EGFR; heparin-binding EGF-like growth factor, betacellulin and epiregulin which bind to both EGFR and ErbB4 [2]; and the neuregulins/heregulins (HRGs), which are specific for ErbB3 and ErbB4 [3]. Although ErbB2 is an orphan receptor with no ligand described to date, it is the preferred dimerzation partner of the other ErbB family members, acting as a potentiator of signalling and highlighting the importance of heterodimerzation within the ErbB family [3-6].

EGF and HRG can activate many intracellular signalling cascades and appear to exert distinct biological functions that depend on the nature of the receptor complexes induced. Although there is major overlap in the signalling pathways activated by ErbB receptors, specific family members can preferentially modulate distinct pathways. For instance, while all ErbB receptors activate the MAPK pathway via Shc and/or Grb2, ErbB3 is the most potent activator of PI3K signalling due to its multiple binding sites for the p85 regulatory subunit of PI3K [7,8]. In contrast, Eps15 and Cbl are both EGFR-specific substrates involved in receptor down-regulation [9,10]. The relative expression of each ErbB receptor influences the cellular response to their ligands. For example, cells expressing high levels of ErbB2 show a greater response to HRG and ErbB3 shows higher affinity for HRG when co-expressed with ErbB2 [11]. This preferential cooperativity extends to oncogenic transformation, with ErbB2-ErbB3 heterodimers reported as the most potent signalling activators [12,13]. Importantly, the aberrant expression and/or activation of ErbB family members have been reported in a number of different tumour types. In particular, there is an extensive literature on the role of ErbB receptors in breast cancer. ErbB2 is overexpressed in 25-30% of all breast cancers due to gene amplification, and is correlated with disease progression, advanced tumour stage, decreased survival, poor response to therapy and metastasis [14,15]. Such poor prognosis is a likely reflection of the biological effects of ErbB2 overexpression, including increased cellular proliferation, anti-apoptosis, cell invasiveness and promotion of angiogenesis. The ErbB receptors have consequently become targets for specific anti-cancer therapies [16-20]. Indeed, one of these therapies, herceptin (trastuzumab), a monoclonal antibody against the extracellular domain of ErbB2, has shown significant clinical benefit for patients with ErbB2-positive breast cancers. Indeed, the combined results of several clinical trials have shown that the addition of 1 year of trastuzumab to adjuvant chemotherapy significantly improves disease-free survival by 33%-52% [21]. Despite this, less than 35% of patients respond to trastuzumab as a single agent and those who initially respond well generally acquire resistance within a year (reviewed in [22]). These data suggest that ErbB2 overexpression alone is not a reliable predictor of therapeutic outcome and that additional factors are involved. Thus, the identification and characterization of genes associated with ErbB2 overexpression would be beneficial, in order to better define the molecular mechanisms involved in ErbB2-dependent transformation and to identify novel drug targets.

Recently, much effort has been put into tumour expression profiling in an attempt to characterize the genes involved in malignant transformation. Microarray analysis has been reported to successfully predict estrogen receptor and lymph-node status of breast cancer [23,24], to distinguish between cancers associated with BRCA1 or BRCA2 mutations [25] and to identify subclasses of breast cancer and predict outcome based on gene expression patterns [23,26-29]. Although these approaches are useful for identifying diagnostic and prognostic markers, few microarray studies have examined ligand-induced signalling events involved in transformation. The aim of this study was to use microarray analysis to investigate ErbB ligand-induced transcriptional responses and diversification of signalling events downstream of ErbB receptors in a human mammary luminal epithelial cell (HMLEC) model. This model comprises an SV40 large T antigen-immortalized HMLEC parental cell line derived from flow-sorted cells from reduction mammoplasty material and a derivative clone stably overexpressing ErbB2 [30,31]. The cells require serum for proliferation, the removal of which leads to loss of viability. In the absence of serum, treatment with the ErbB-specific ligands HRGβ1 or EGF can support proliferation and survival, with the ErbB2-overexpressing cells displaying increased rates of proliferation, anchorage-independent growth and enhanced mitogenic signalling compared to the parental line [31,32]. In the present study, cells were serum-starved and treated with EGF or HRGβ1 over a timecourse to establish long-term HRG- and EGF-specific transcriptional responses to examine diversification of signalling through EGFR and ErbB3 receptors and to assess how ErbB2 overexpression alters these responses.

## Methods

### Cell culture, growth factor stimulation and RNA isolation

The parental HMLEC line HB4a and an ErbB2-overexpressing derivative C3.6 have been previously described [31,32]. Cells were cultured in RPMI-1640 media supplemented with 10% fetal calf serum (FCS), 2 mM glutamine, 100 IU/ml penicillin, 100 μg/ml streptomycin, 5 μg/ml hydrocortisone and 5 μg/ml insulin (both Sigma) at 37°C in a 10% CO_2 _humidified incubator. Before stimulation, HB4a and C3.6 cells were starved of GFs for 48 h in RPMI-1640 media with 0.1% FCS, 100 IU/ml penicillin, 100 μg/ml streptomycin and 5 μg/ml hydrocortisone. Cells were then treated with 1 nM EGF or 1 nM HRGβ1 (HRG hereafter) (both R&D Systems) for 4 h, 18 h and 24 h prior to RNA isolation. Two plates were prepared for control (serum-starved) cells and for each time point. Total RNA was isolated using TRIZOL™ reagent (Invitrogen Life Technologies) according to the manufacturer's protocol. Each plate generated two samples of total RNA for reciprocal labelling, resulting in a total of four replicates for microarray experiments. Serum starved cells were also treated with 25 ng/mL IGF1 for the indicated times. SKBR3 cells were maintained in tissue culture flasks containing DMEM/F-12 medium supplemented with 10% (v/v) FCS, 100 μg/mL streptomycin and 100 IU/mL penicillin (Gibco-Invitrogen Corp) in a humidified incubator at 37˚C with 5% CO_2_.

### Microarray experimental design

Hver 1.3.1 arrays used in this study were obtained from the Wellcome Trust Sanger Institute. Each microarray contains a redundant set of 9932 PCR-derived, sequence verified cDNA clones representing around 6,000 genes. 25 μg of total RNA was used to produce labeled cDNA by anchored oligo(dT)-primed reverse transcription with Superscript II Reverse Transcriptase (Invitrogen Life Technologies) in the presence of Cy3- or Cy5-dUTP (Amersham Pharmacia). Unincorporated Cy dye was removed using Autosequ-50 Columns (Amersham Pharmacia) and repetitive DNA sequences were blocked by co-precipitation of labeled cDNA with 8 μg Cot1 (Boehringer Mannheim) and 8 μg poly(dA) DNA (Sigma). The labeled cDNA pellet was re-suspended in hybridization buffer (4 × SSC, 5× Denhardt's solution, 50 mM Tris-HCl pH 7.6, 0.1% sarkosyl, 49% formamide) and hybridized onto arrays at 47°C overnight. Slides were washed twice in 2 × SSC, four times in 0.1 × SSC plus 0.1% SDS, twice in 0.1 × SSC and then dried by centrifugation before scanning. All samples were co-hybridized to a common standard reference comprised of total RNA pooled from cell line BT474 and two grade III invasive ductal breast carcinomas (gift of Dr Alan Mackay, ICR, London), allowing cross-comparison of multiple experimental conditions. A "dye-flip" approach was used to minimize dye-specific bias, where two biological replicates were labeled with Cy3 and hybridized with Cy5-labelled reference and vice versa (four replicates). For the 14 experimental conditions (2 cell lines, 2 GFs and 3 time points plus untreated), a total of 56 hybridizations were thus performed.

### Data normalization, filtering and analysis

Slides were scanned using ScanArray 4000XL and spots quantified using QuantArray v3.0 (both Packard BioChip Technology). Cy-dye emission signals were scaled in QuantArray using the median intensity of each channel, and any visible hybridization artifacts were flagged and recorded as absent during data filtering and analysis. Data was background subtracted and exported to GeneSpring v6.1 (Silicon Genetics) for normalization. The fluorescence intensity ratio between each sample and the co-hybridized reference (sample/ref) was calculated and represents the expression level for a given probe on each individual replicate. The dataset was then normalized using the intensity-dependent LOWESS regression technique [33]. Normalized raw data and experimental details were processed to conform with Minimum Information About a Microarray Experiment (MIAME) guidelines and are deposited in the Array Express (http://www.ebi.ac.uk/arrayexpress) repository (accession number E-TABM-106). Genes were then filtered using the excel add-in SAM (Significant Analysis of Microarray) [34] to identify genes showing significant changes in expression by assigning a score on the basis of change in gene expression relative to the standard deviation of repeated measurements. A false discovery rate threshold of 3% was used as the cut-off to report differentially regulated genes. Averaged values for each of the 14 experimental conditions were then compared to identify genes that were up- or down-regulated generating a list of 775 genes that changed significantly between two or more experimental conditions. TIGR MeV software v2.2 (The Institute for Genomic Research) was used for clustering analysis of the 775 genes using two sets of average ratios: i) the HB4a/C3.6 ratio was taken at each time point for ErbB2-dependent changes in gene expression, and ii) the T*/T0 ratio was taken in each cell line for GF-dependent changes in gene expression. Values were log2 transformed, loaded into TIGR MeV for average-linkage hierarchical clustering using Euclidian Distance and for *k*-means clustering using 4 (*k*) groups. Genes in each group were then subjected to hierarchical clustering.

### Semi-quantitative Real Time-PCR

Samples were generated by reverse transcription of 2.5 μg of total RNA using Superscript II Reverse Transcriptase (Invitrogen Life Technologies) and random hexamer primers (Applied Biosystems). cDNAs were then treated with RNase H (Invitrogen Life Technologies) to eliminate RNA contamination. Real Time-PCR (qRT-PCR) was performed using the Assay-on-Demand system (Applied Biosystems) according to the manufacturer's protocol. Assay IDs were: Hs99999905_m1 (GAPDH); Hs99999901_s1 (18S); Hs00155832_m1 (AREG); Hs00426287_m1 (IGFBP3); Hs00192713_m1 (G1P2); Hs00175188_m1 (CTSC); Hs00196051_m1 (ISGF3G); Hs00242943_m1 (OAS1); Hs00195584_m1 (S100P); Hs00602835_s1 (SFN); Hs00173626_m1 (VEGF); Hs00185574_m1 (VIL2); Hs00185584_m1(VIM); Hs00170299_m1 (ZYX). Briefly, primer and probe mix was added to PCR Master Mix with 1 μL of cDNA per 50 μL reaction. Sample fluorescence emission was recorded for each cycle on an ABI7700 Sequence Detection System. Cycling conditions were as follows: initial enzyme activation step at 95°C for 10 min and then 39 repeating cycles of 95°C for 10 sec and 60°C for 1 min. Serial dilution experiments of each primer against the endogenous control were prepared to test for amplification efficiency. For primers whose amplification efficiencies were similar to that of the endogenous control (slope of the graph ΔCt vs. log dilutions ≤0.1) the ΔCt method was used, where the equation 2^-ΔΔCt ^determines the amount of a target relative to a calibrator sample. If amplification efficiencies were not similar, the standard curve method was used for relative quantitation of target gene expression. Further information on both of these methods can be found on User Bulletin3 on the ABI website.

### Immunoblotting

Freshly treated cells were lysed in NP40 lysis buffer (50 mM HEPES pH 7.4, 150 mM NaCl, 1% NP40, 1 mM EDTA) with protease and phosphatase inhibitors: pepstatin A (1 μg/mL), leupeptin (1 μg/mL), AEBSF (100 μg/mL), aprotinin (17 μg/mL), sodium orthovanadate (2 mM), okadaic acid (1 μM), fenvalerate (5 μM) and bpV (5 μM). Protein concentration was determined using a Bradford assay and equal amounts of protein used for SDS-PAGE and immunoblotting with commercially-available antibodies (Additional file 1). Antibodies were detected with appropriate HRP-conjugated secondary antibodies and detected by enhanced chemiluminescence (Perkin Elmer). All membranes were reprobed for beta-actin and densitometry performed on all bands using a GS-800 Calibrated Densitometer and QuantityOne software (both BioRad) with local background subtraction. Intensities for each band were then normalized to the actin band in that lane. Normalized values were averaged from 3-5 independent blots and plotted using the standard deviation as the error.

### Small interfering RNA (siRNA) reverse transfection

HB4a, C3.6, or SKBR3 cells were withdrawn from antibiotics for a minimum of 2 hrs and subsequently transfected with siRNA pools targeting ErbB2, IGFBP3 or non-targeting scrambled control siRNA (Dharmacon RNA Technologies); the ON-TARGET plus non-targeting control siRNA pool was used in invasion, proliferation and stimulation assays, whilst the #2 ON-TARGET plus non-targeting control siRNA was used in anchorage-independence growth assays. Reverse transfection was performed in 6-well plates according to the manufacturer's instructions using Lipofectamine™ RNAi Max (Invitrogen) and diluting the siRNA with Opti-Mem^® ^reduced serum medium (Invitrogen). A final concentration of 50 nM of siRNA was typically used to transfect 2.5 × 10^5 ^cells per well (or 1.5 × 10^5 ^for siIGFBP3 knockdown in SKBR3) which were then maintained in their normal growth medium. Cells were typically harvested 96 hrs post-transfection in 200 μL of NP40 lysis buffer and expression knockdown confirmed by western blotting as described.

### Invasion assays

Transfected SKBR3 cells were subjected to a matrigel-based invasion assay utilizing a 24-well BD Biocoat™ Tumour Invasion Assay System (BD Biosciences) according to the manufacturer's instructions. At 96 hours post-transfection, cells were harvested, counted and plated at 1 × 10^5 ^cells/chamber in DMEM/F-12 medium supplemented with 0.1% (v/v) FCS, 100 μg/mL streptomycin and 100 IU/mL penicillin. The lower chamber contained a chemo-attractant of DMEM/F-12 medium supplemented with 10% (v/v) FCS. Invaded cells on the underside of the membrane were fixed and stained after 72 hours in a 99% methanol, 1% crystal violet solution and counted under a bright field microscope with the Image J software. Experiments were performed in triplicate for each siRNA and cells in 5 fields per membrane were counted.

### Proliferation assays

Transfected cells were harvested after 96 hrs, counted and plated at 3 × 10^3 ^(SKBR3) and 5 × 10^3 ^cells/well (HMLEC) into 96-well plates in DMEM/F-12 medium supplemented with 10% (v/v) FCS, 100 μg/mL streptomycin and 100 IU/mL penicillin with 5 replicates per condition. The number of viable cells was ascertained utilizing a MTT (3-(4, 5-dimehylthiazol-2-yl)-2-5-dyphennyltetrazolium bromide) assay after 48 hrs. For this, cells were incubated with 50 μL/well of 1 mg/mL MTT which is converted to purple formazan crystals by viable cells. After 5 hr crystals were solubilised in 100 μL of DMSO, shaken at room temperature for 10 min and the absorbance measured at 540 nm using a microtitre plate spectrophotometer.

### Anchorage-independence growth assays

Transfected SKBR3 cells were harvested after 96 hrs, counted and re-suspended in DMEM/F-12 medium supplemented with 10% (v/v) FCS, 100 μg/mL streptomycin, 100 IU/mL penicillin and 10% (v/v) of a 10 mg/mL bacto-peptone solution which contained 3.3% (v/v) noble agar (both Sigma). Cells were plated at 2 × 10^4 ^cells/well into 6-well plates containing DMEM/F-12 medium supplemented with 10% (v/v) FCS, 100 μg/mL streptomycin, 100 IU/mL penicillin and 10% (v/v) of a 10 mg/mL bacto-peptone solution with 6% (v/v) noble agar. Colonies were fixed and stained after 14 days with 1 mg/mL p-iodotertazolium violet (Sigma) prepared in absolute methanol and counted using the Image J software. Experiments were performed in triplicate and 5 fields were counted per plate.

## Results

### ErbB2 and growth factor cDNA microarray analysis

Our aim was to identify time-dependent and growth factor-specific changes in gene expression associated with long-term EGF and HRGβ1 (hereafter HRG) stimulation of a model HMLEC system. The concentrations of growth factor used (1 nM) were selected based on minimum concentrations required for maximal activation of the ERK1/2 and Akt pathways as measured by western blotting with phospho-specific antibodies (data not shown). We also wanted to assess how such gene expression changes are affected by ErbB2 overexpression in order to understand how ErbB2 contributes to signalling events associated with breast epithelial cell transformation. Microarray experiments were thus carried out using a previously described ErbB2-overexpressing HMLEC system [31,32]. Serum-starved HB4a parental cells and ErbB2-overexpressing C3.6 cells were stimulated with EGF or HRG for 4 h, 18 h and 24 h or left unstimulated (0 h) prior to microarray analysis of mRNA levels of 9,932 probes, representing ~6,000 genes. These timepoints were chosen to assess the medium-term effects on gene expression that occur within the doubling time of the cells.

**Figure 1:**
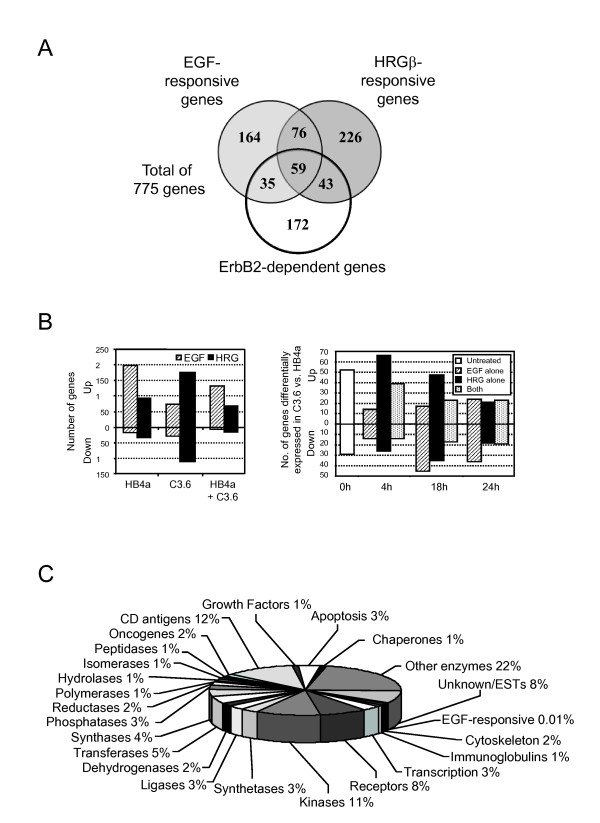
**Numbers and functions of differentially expressed genes**. A. Venn diagram showing the distribution of the 775 genes found to be significantly differentially expressed and their co-regulation by EGF, HRG and ErbB2. B. Distribution of up- or down-regulated genes by cell line and growth factor (left) and in C3.6 vs. HB4a over time (right). C. Distribution of functional classes of differentially regulated genes based on GO terms for molecular function.

Four replicates of each sample were co-hybridized to chips with a common RNA reference allowing direct comparison of gene expression across all experimental conditions: time, GF and cell line. The excel add-in software SAM (Significance Analysis of Microarray) [34] was used to perform pair-wise comparisons of all conditions, identifying 1,995 significant changes, representing 775 genes whose expressions were either responsive to EGF and/or HRG, genes differentially regulated by ErbB2 overexpression, or both. Of these, 145 genes occurred more than once where probes on the arrays corresponded to different sequences of the same gene. In general, these duplicates and triplicates displayed similar patterns of expression, increasing confidence in the observed changes. Full gene lists grouped by responsiveness are available in Additional file 2. Fig 1A and 1B show the number of genes and their overlapping responses. There were a greater number of genes up- or down-regulated by HRG in the C3.6 cells than EGF, whilst EGF was generally more potent at inducing expression in the HB4a parental cells. Time-dependent changes in the response to each GF were also apparent between cell lines, indicating that ErbB2 overexpression alters the kinetics of downstream signalling events. Functional classification of the 775 genes revealed a wide range of gene families, with kinases, CD antigens and receptors featuring prominently (Fig. 1C). There were surprisingly few known EGF-responsive genes, although this may reflect the timepoints employed and/or insufficient database annotation. Fifty-two genes had no functional annotation and represent novel genes that warrant further study.

**Figure 2:**
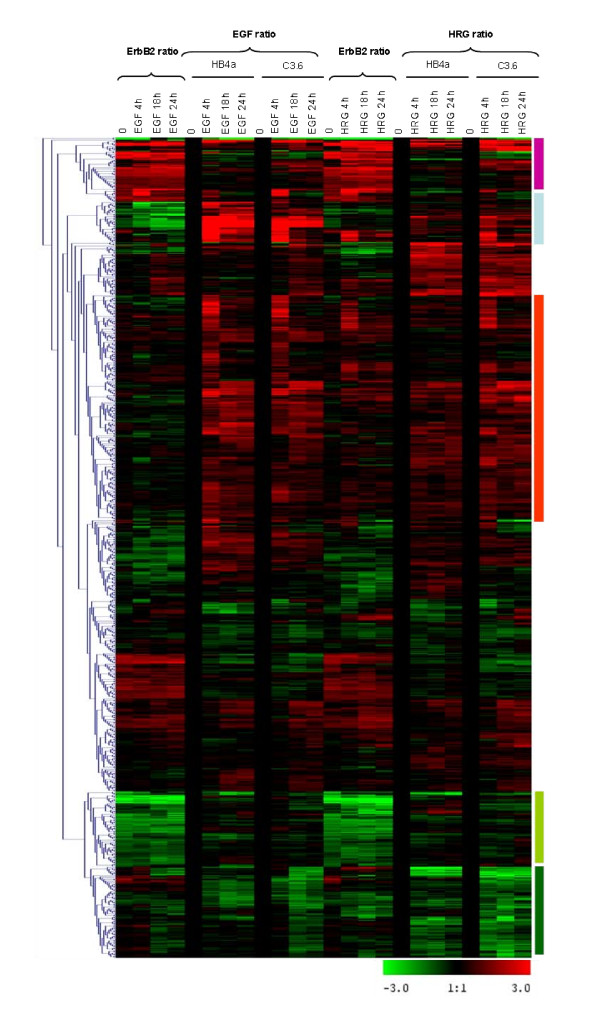
**Hierarchical clustering of 775 differentially expressed genes**. Ratios of normalized values were used to show relative gene expression in two ways: (i) the EGF or HRG ratio (T*/T0) representing relative gene expression at each timepoint in relation to the untreated control, measuring the response to each ligand in HB4a and C3.6 cells separately, and (ii) the ErbB2 ratio (C3.6/HB4a) representing relative gene expression in C3.6 vs. HB4a at each time point, identifying genes whose expression are affected by ErbB2. Ratios were log2 transformed and loaded into TIGR MeV software v2.2 (Institute for Genomic Research) and unsupervised, average-linkage hierarchical clustering performed. Clusters indicated by light green and pink bands (on right) show genes whose expressions were altered by ErbB2 overexpression, but were similarly up- or down-regulated by EGF and HRG; the light blue cluster shows genes down-regulated by ErbB2, but up-regulated by EGF; the dark green cluster shows genes that were down-regulated by both growth factors and the red cluster shows genes that were transiently-induced in all treatments except in HRG-treated HB4a cells.

**Figure 3:**
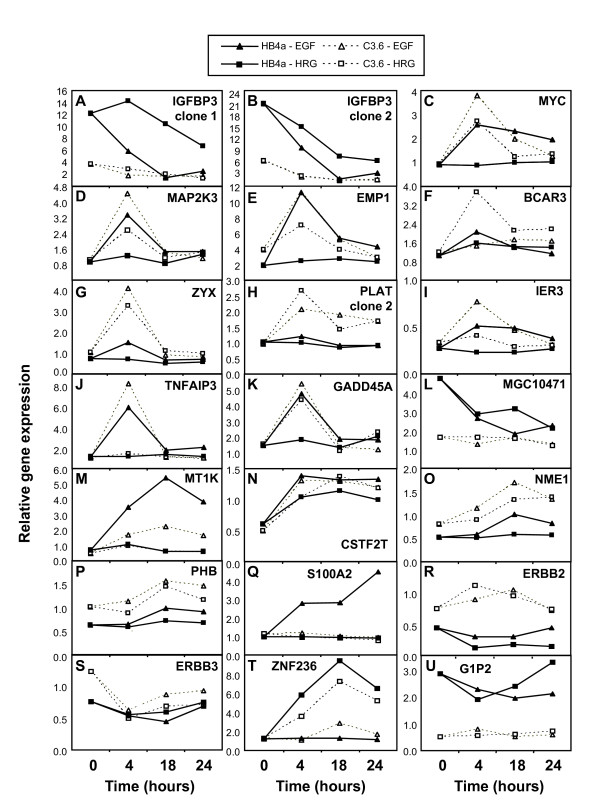
**Examples of growth factor and ErbB2-dependent differential gene expression**. Plots of relative gene expression (averaged (n = 4) normalised fluorescence intensity) versus time are shown for 20 genes. Data for both microarray clones are shown for IGFBP3. Genes showing a change in expression following growth factor treatment and significantly different between the cell lines were: IGFBP3_1 (1.18-fold increase in HB4a versus 1.3-fold decrease in C3.6 with EGF (0 to 4 hr); MYC (1.05-fold decrease in HB4a versus 3.09-fold increase in C3.6 with HRG (0 to 4 hr); MAP2K3 (1.39-fold increase in HB4a versus 2.49-fold increase in C3.6 with HRG (0 to 4 hr); BCAR3 (1.52-fold increase in HB4a versus 3.18-fold increase in C3.6 with HRG (0 to 4 hr); ZYX (1.05-fold decrease in HB4a versus 3.17-fold increase in C3.6 with HRG (0 to 4 hr); PLAT2 (1.18-fold increase in HB4a versus 2.13-fold increase in C3.6 with EGF (0 to 4 hr) and 1.01-fold increase in HB4a versus 2.76-fold increase in C3.6 with HRG (0 to 4 hr); TNFAIP3 (4.98-fold increase in HB4a versus 7.74-fold increase in C3.6 with EGF (0 to 4 hr); GADD45A (1.26-fold increase in HB4a versus 2.82-fold increase in C3.6 with HRG (0 to 4 hr); S100A2 (2.88-fold increase in HB4a versus 1.02-fold increase in C3.6 with EGF (0 to 4 hr); ERBB2 (3.13-fold decrease in HB4a versus 1.48-fold increase in C3.6 with HRG (0 to 4 hr); ZNF236 (4.85-fold increase in HB4a versus 2.83-fold increase in C3.6 with HRG (0 to 4 hr).

Ratios of normalized values were next used to show relative gene expression in two ways: (i) the EGF or HRG ratio (T*/T0) representing gene expression at each timepoint relative to untreated control for both cell lines, and (ii) the ErbB2 ratio (C3.6/HB4a) representing relative gene expression in C3.6 versus HB4a cells at each timepoint. Ratios were used for unsupervised hierarchical clustering of all 775 genes (Fig. 2). Although no striking functional clusters were apparent, there were several interesting features. One group of genes displayed altered expression correlating with ErbB2, but displayed similar levels of up- or down-regulation in response to both growth factors (light green and pink bands, Fig. 2). A second group of genes was down-regulated by ErbB2, but potently up-regulated by EGF treatment (light blue band). A third large group of genes displayed transient induction (up at 4 h only) in all treatments, but less so with HRG in the HB4a cells (red band). A fourth group of genes were down-regulated in response to both GFs (dark green band). Finally, two features clustered away from the other 773 genes (top of cluster in Fig. 2) and represented IGFBP3, a putative regulator of insulin-like growth factor (IGF)-dependent and independent proliferation, differentiation and survival. IGFBP3 expression was strongly suppressed by ErbB2 overexpression and down-regulated by both GFs (Fig. 3A &3B).

### Growth factor-induced genes augmented by ErbB2 overexpression

Genes transiently induced by both GFs and whose expressions were induced more strongly in the ErbB2-overexpressing cells are of particular interest in ErbB2-dependent cell transformation. These genes included transcription factors MYC (Fig. 3C), ZFP36L1, ZFP36L2, FOSL1 and ATF4/CREB2, growth factors VEGF and PDGFB and signalling kinases LYN, MAP2K1/MEK1 and MAP2K3/MEK3 (Fig. 3D). Also in this group, were the MYC-induced glycoprotein EMP1, which showed a similar pattern of expression to MYC (Fig. 3E), and BCAR3 (Fig. 3F), a novel SH2 and GEF domain-containing gene. A number of genes associated with cytoskeletal organization and adhesion were also induced more robustly in the ErbB2 overexpressing cells, particularly in response to HRG. These genes included zyxin (ZYX) (Fig. 3G), transgelin (TAGLN), thrombospondin 1 (THBS1), vinculin (VCL), calponin 3 (CNN3), villin 2/ezrin (VIL2), myosin 1E (MYO1E), stathmin 3 (STMN3), Crk-associated substrate-related protein (NEDD9/CASL), ladinin 1 (LAD1) and integrin α2 and α3 (ITGA2 and ITGA3). Members of the plasminogen activator system; tissue-type plasminogen activator (PLAT) (Fig 3H), urokinase-type plasminogen activator receptor (PLAUR), plasminogen activator inhibitor 1 (PAI1/SERPINE1) and the plasminogen and PLAT co-receptor annexin A2 (ANXA2), were also present in this group. The anti-apoptotic genes IER3 and TNFAIP3 (Fig. 3I &3J) were also more highly induced in the ErbB2-overexpressing cells, as were the poorly characterized genes S100P, CSRP1, HPCAL1 and SMAP, identifying them as potential effectors of ErbB signalling. Finally, and perhaps surprisingly, the genotoxic stress-induced growth arrest gene GADD45A (Fig. 3K) and the MAPK phosphatases DUSP1/MKP1 and DUSP5 were both induced by growth factor treatment.

### Growth factor-induced gene expression changes

**Figure 4:**
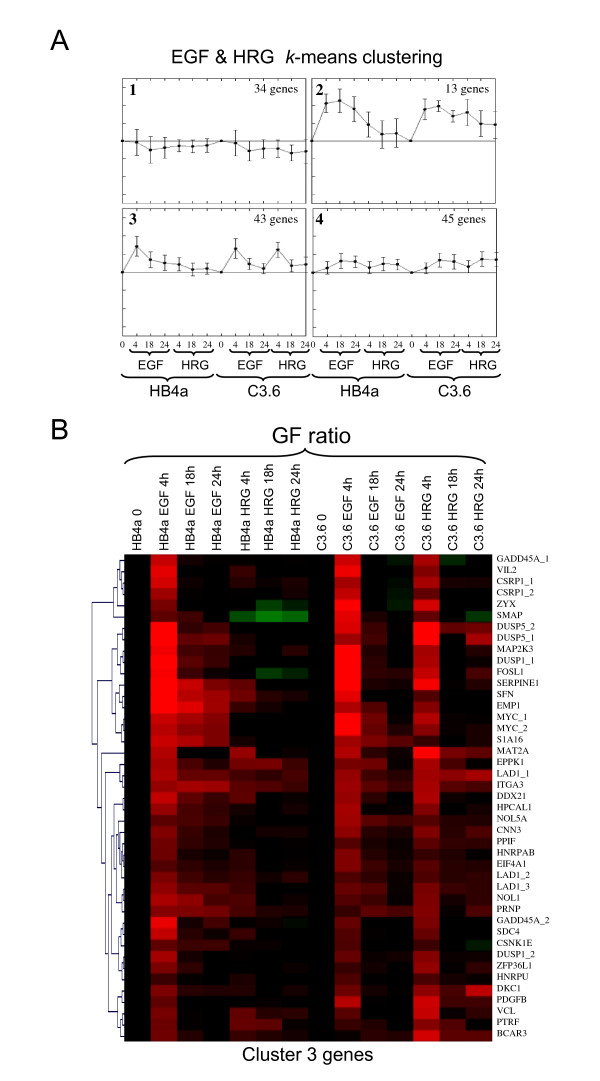
***K*-means clustering and hierarchical clustering of EGF and HRG-responsive genes**. A. For *k*-means clustering in TIGR MeV software v2.2 (Institute for Genomic Research), growth factor-responsive genes from SAM were grouped separately and sub-divided into a user-defined number (*k *= 4) of groups. Plots show the four groups as average expression patterns for genes regulated by both growth factors. B. Group 3 genes which were transiently induced by both growth factors except by HRG in HB4a cells were subjected to hierarchical clustering as above.

In order to analyze EGF and HRG-specific changes in gene expression regardless of ErbB2 level, three gene lists were generated according to the Venn diagram in Fig. 1A: (i) EGF-responsive (199 genes); (ii) HRG-responsive (269 genes); and (iii) genes responsive to both (135 genes). Genes in each list were grouped by *k*-means clustering into 4 clusters displaying similar patterns of expression. Fig 4A shows the average expression patterns for genes regulated by both GFs and reveals that a considerable number were less potently induced (or not induced) by HRG in the HB4a cells (Fig. 4A; clusters 2 & 3). Of the 13 genes in cluster 2 (Figs. 4A & Additional file 3), six were clones of the metallothionein (MT) family of cysteine-rich, heavy metal-binding proteins and were potently induced by EGF (Fig. 3M). This cluster also included cytoskeletal components keratin 6 (KRT6D/B), transgelin (TAGLN) and coactosin-like 1 (COTL1) and a novel polyadenylation protein variant tauCstF-64 (CSTF2T) (Fig. 3N). Cluster 3 genes (Fig. 4B) included SFN, a negative regulator of G2/M progression and the adhesion-related genes villin 2/ezrin (VIL2), syndecan 4 (SDC4), integrin-α3 (ITGA3), integrin-β1 (ITGB1) laminin-α3 (LAMA3), paxillin (PXN) and vinculin (VCL). Clusters 1 and 4 showed a more similar pattern of regulation in the two cell lines and their regulation is therefore less likely to be dependent upon ErbB2 signalling. Cluster 1 genes were down-regulated in response to GF treatment and included genes involved in the response to oxidative stress (PRDX5, GSTP1, MGST3, TXNIP and ALDH1A3). Notably, a gene of unknown function (MGC10471/CCDC130) was potently repressed by both GFs in HB4a cells, and was constitutively down-regulated in C3.6 cells, similar to IGFBP3 (Fig. 3L). Such novel genes are of particular interest since they may be components of as yet uncharacterized GF-dependent pathways. wfdc2 Cluster 4 genes were moderately up-regulated by EGF and HRG, and included genes with reported roles in proliferation and tumour suppression, such as MYBL2, FOXM1, NME1 (Fig. 3O) and PHB (Fig. 3P).

**Figure 5:**
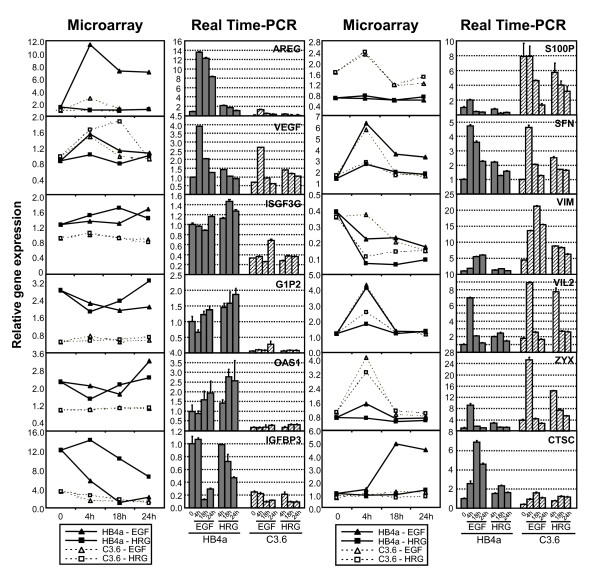
**Comparison of microarray and real time RT-PCR data**. Real time RT-PCR was performed on 12 target genes using Applied Biosystems' Assay-on-Demand and relative gene expression calculated using the ΔCt or standard curve method (see Methods for details).

Genes induced specifically by EGF included C14orf31, CDH3, CAV1, ACTB, CTNNAL1, PTPN1, S100A11, TIMP1, TMSB10 and TXN, linking them to signalling through EGFR-containing dimers. A group of EGF-specific genes were induced almost exclusively in HB4a cells and included the EGFR ligand AREG (Fig. 5), the cysteine protease cathepsin C (CTSC) (Fig. 5), the Ca^2+^-binding protein S100A2 (Fig. 3Q) and hypoxia inducible factor 2A (HIF2A), a transcription factor involved in the induction of oxygen-regulated genes such as VEGF (see above). There were no genes exclusively down-regulated by EGF, although IGFBP3 and the transmembrane glycoprotein MUC1 showed a stronger response to EGF than HRG. There were significantly more genes responsive to HRG, and although the inductions were generally weaker, their expressions were often sustained. ERBB2 and ERBB3 (Fig. 3R &3S) were among the genes down-regulated by HRG, suggesting a feedback mechanism through regulated transcription. Noticeably, ERBB2 and ERBB3 gene expression was higher in the C3.6 cells, in accordance with previously established protein levels [32]. Genes potently induced by HRG compared to EGF included metabolic enzymes, transcription factors (MADH4 and STAT1), the cell cycle inhibitor CDKN1A/p21CIP, the protease inhibitor SLPI, fibronectin 1 (FN1), keratin 15 (KRT15) and the poorly characterized NUP214 and zinc finger-containing gene (ZNF236) (Fig. 3T). Figures of *K*-means clusters of genes regulated by either EGF or HRG can be found in Additional files 4 and 5.

### ErbB2-dependent gene expression

A group of genes displayed differential expression between the cell lines, but were either unresponsive to GF treatment or responded similarly in the two cell lines (Table 1). These included several metabolic enzymes that were more highly expressed in the C3.6 cells, and eight known interferon-stimulated genes (ISGs) that displayed reduced expression. The ISGs included the ubiquitin-like modifier G1P2/ISG15, which was the most suppressed gene in the dataset (Fig. 3U), and ISGF3G/p48/IRF9, the third component of a STAT1and STAT2-containing transcription factor complex that controls interferon (IFN)-mediated gene expression. Finally, several genes of poorly-defined or unknown function (AGR2, PSCA, PKM2, NME1, CPNE3, LCP1, S100P, SERF2 and LOC402057) were constitutively up-regulated in the C3.6 cells (Table 1) and may represent novel markers of ErbB2-dependent transformation.

**Table 1:** Genes displaying differential expression between HB4a and C3.6 cell lines.

Symbol	Ensembl Number and Description	GO Biological Process	Fold Change (at T0)
*ALDH1A3	ENSG00000184254:ALDEHYDE DEHYDROGENASE 6	GO:0006629:lipid metabolism	5.45

KRT15	ENSG00000171346:KERATIN, TYPE I CYTOSKELETAL 15	GO:0008544:epidermal differentiation	4.66

*AGR2	ENSG00000106541:ANTERIOR GRADIENT 2	Unknown	4.00

NCKAP1	ENSG00000061676:NCK-ASSOCIATED PROTEIN 1 (NAP 1)	GO:0006915:apoptosis	3.87

COX6C	ENSG00000164919:CYTOCHROME C OXIDASE POLYPEPTIDE VIC	GO:0006091:energy pathways	3.37

PSCA	ENSG00000167653:PROSTATE STEM CELL ANTIGEN PRECURSOR	Unknown	2.94

*KRT13	ENSG00000171401:KERATIN, TYPE I CYTOSKELETAL 13	GO:0008544:epidermal differentiation	2.69

YWHAZ	ENSG00000164924:14-3-3 PROTEIN ZETA/DELTA	GO:0007165:signal transduction	2.47

*S100P	ENSG00000163993:S-100P PROTEIN	Unknown	2.45

*PKM2	ENSG00000067225:PYRUVATE KINASE, MUSCLE	GO:0006096:glycolysis	2.41

TRAM1	ENSG00000067167:TRAM PROTEIN	GO:0006605:protein targeting	2.38

ATP5L	ENSG00000167283:ATP SYNTHASE G CHAIN, MITOCHONDRIAL	GO:0015992:proton transport; GO:0006754:ATP biosynthesis	2.26

SLC7A7	ENSG00000155465:Y+L AMINO ACID TRANSPORTER 1	GO:0006832:small molecule transport	2.25

SSBP1	ENSG00000106028:SINGLE-STRANDED DNA-BINDING PROTEIN	GO:0006260:DNA replication	2.25

SERF2	ENSG00000140264:SMALL EDRK-RICH FACTOR 2	Unknown	2.21

NEDD9	ENSG00000111859:ENHANCER OF FILAMENTATION 1 (HEF1)	GO:0007155:cell adhesion; GO:0000074:regulation of cell cycle	2.18

SCAMP2	ENSG00000140497:SECRETORY CARRIER-ASSOCIATED MEMBRANE PROTEIN 2	GO:0006886:intracellular protein transport	2.12

EMP1	ENSG00000134531:EPITHELIAL MEMBRANE PROTEIN-1	GO:0007048:oncogenesis;GO:0008283:cell proliferation	2.10

*LCP1	ENSG00000136167:L-PLASTIN	Unknown	2.09

MGST1	ENSG00000008394:MICROSOMAL GLUTATHIONE S-TRANSFERASE 1	GO:0032496: response to lipopolysaccharide	2.06

DUT	ENSG00000128951:DEOXYURIDINE 5'-TRIPHOSPHATE NUCLEOTIDOHYDROLASE	GO:0006260:DNA replication	2.05

DDX5	ENSG00000108654:PROBABLE RNA-DEPENDENT HELICASE P68	GO:0016049:cell growth	2.00

SRP14	ENSG00000140319:SIGNAL RECOGNITION PARTICLE 14 KDA PROTEIN	GO:0006605:protein targeting	1.98

*ANXA2	ENSG00000183059:ANNEXIN II (LIPOCORTIN II)	GO:0001501:skeletal development	1.95

PBP	ENSG00000089220:PHOSPHATIDYLETHANOLAMINE-BINDING PROTEIN	Unknown	1.80

ATP5G3	ENSG00000154518:ATP SYNTHASE LIPID-BINDING PROTEIN	GO:0006091:energy pathways; GO:0015992:proton transport	1.78

SRI	ENSG00000075142:SORCIN (22 KDA PROTEIN)	GO:0007517:muscle development	1.77

RPL17	ENSG00000141618:60S RIBOSOMAL PROTEIN L17	GO:0006412:protein biosynthesis	1.71

*ERBB2	ENSG00000141736:V-ERBB2 ERYTHROBLASTIC LEUKEMIA VIRAL ONCOGENE HOMOLOG 2	GO:0007169:transmembrane receptor protein tyrosine kinase signaling	1.67

*AKR1B1	ENSG00000085662:ALDOSE REDUCTASE	GO:0005975:carbohydrate metabolism	1.66

ATP6V1F	ENSG00000128524:VACUOLAR ATP SYNTHASE SUBUNIT F	GO:0015992:proton transport; GO:0006754:ATP biosynthesis	1.66

ATP5G1	ENSG00000159199:ATP SYNTHASE LIPID-BINDING PROTEIN	GO:0015992:proton transport	1.64

UBN1	ENSG00000118900:UBINUCLEIN 1	GO:0016568:chromatin modification	1.63

*ERBB3	ENSG00000065361:V-ERBB2 ERYTHROBLASTIC LEUKEMIA VIRAL ONCOGENE HOMOLOG 3	GO:0007169:transmembrane receptor protein tyrosine kinase signaling	1.62

FTH1	ENSG00000167996:FERRITIN HEAVY CHAIN	GO:0008283:cell proliferation; GO:0006826:iron transport	1.62

ST14	ENSG00000149418:SUPPRESSOR OF TUMORIGENICITY 14	GO:0006508:proteolysis and peptidolysis	1.62

*PHB	ENSG00000167085:PROHIBITIN	GO:0008151:cell growth and/or maintenance	1.59

CLTC	ENSG00000141367:CLATHRIN HEAVY CHAIN 1	GO:0006886:intracellular protein transport	1.51

APLP2	ENSG00000084234:AMYLOID-LIKE PROTEIN 2 PRECURSOR	GO:0007186:G-protein coupled receptor protein signaling pathway	1.48

HSBP1	ENSG00000166530:HEAT SHOCK FACTOR BINDING PROTEIN 1	GO:0000122:negative regulation of transcription from Pol II promoter	1.48

*NME1	ENSG00000239672:NON-METASTATIC CELLS 1	GO:0045786:negative regulation of cell cycle; GO:0009142: NTP biosynthesis	1.47

*CPNE3	ENSG00000085719:COPINE III	Unknown	1.46

*ISGF3G †	ENSG00000213928:INTEREFERON REGULATORY FACTOR 9	GO:0006355:regulation of transcription; GO:0006955: immune response	0.73

*USP14 †	ENSG00000101557:UBIQUITIN SPECIFIC PEPTIDASE 14	GO:0006511:ubiquitin-dependent protein catabolism	0.71

SSR4	ENSG00000180879:SIGNAL SEQUENCE RECEPTOR DELTA	GO:0006886:intracellular protein transport	0.69

RAB7	ENSG00000075785:RAS-RELATED PROTEIN RAB-7	GO:0007264:small GTPase mediated signal transduction; GO:0006897:endocytosis	0.69

SHMT2	ENSG00000182199:SERINE HYDROXYMETHYLTRANSFERASE	GO:0006520:amino acid metabolism	0.68

NGFRAP1	ENSG00000166681:NERVE GROWTH FACTOR RECEPTOR ASSOCIATED PROTEIN 1	GO:0007275:development; GO:0006915:apoptosis	0.66

HAT1	ENSG00000128708:HISTONE ACETYLTRANSFERASE 1	GO:0006323:DNA packaging; GO:0006475: internal protein amino acid acetylation	0.65

PPP1CA	ENSG00000172531:SERINE/THREONINE PROTEIN PHOSPHATASE PP1-ALPHA 1 CATALYTIC SUBUNIT	GO:0006470:protein amino acid dephosphorylation	0.65

UBL1	ENSG00000116030:UBIQUITIN-LIKE PROTEIN SMT3C PRECURSOR	GO:0006281:DNA repair	0.65

NPC2	ENSG00000119655:EPIDIDYMAL SECRETORY PROTEIN E1 PRECURSOR	GO:0000004:biological_process unknown	0.65

GMPS	ENSG00000163655:GMP SYNTHASE	GO:0006164:purine nucleotide biosynthesis	0.62

UBE2L6 †	ENSG00000156587:UBIQUITIN-CONJUGATING ENZYME E2L 6	GO:0006512:ubiquitin cycle	0.61

SF3B1	NSG00000115524:SPLICING FACTOR 3B SUBUNIT 1	GO:0006371:mRNA splicing	0.58

ANXA1	ENSG00000135046:ANNEXIN I	GO:0006928:cell motility;GO:0006629:lipid metabolism	0.57

SDC1	ENSG00000115884:SYNDICAN 1	GO:0048627:myoblast development	0.56

FXR1	ENSG00000114416:FRAGILE X MENTAL RETARDATION SYNDROME RELATED PROTEIN 1	GO:0006915:apoptosis	0.56

PLD3	ENSG00000105223:SIMILAR TO VACCINIA VIRUS HINDIII K4L ORF	GO:0008152:metabolism	0.53

RPN1	ENSG00000163902:DOLICHYL-DIPHOSPHOOLIGOSACCHARIDE--PROTEIN GLYCOSYLTRANSFERASE	GO:0006464:protein modification	0.51

*GSTP1 †	ENSG00000084207:GLUTATHIONE S-TRANSFERASE P	GO:0007417:central nervous system development	0.51

HDLBP	ENSG00000115677:VIGILIN (HIGH DENSITY LIPOPROTEIN-BINDING PROTEIN)	GO:0006869:lipid transport; GO:0008203:cholesterol metabolism	0.5

TYMS	ENSG00000176890:THYMIDYLATE SYNTHASE	GO:0006139:nucleobase, nucleoside, nucleotide and nucleic acid metabolism	0.44

WFDC2	ENSG00000101443:MAJOR EPIDIDYMIS-SPECIFIC PROTEIN E4 PRECURSOR	GO:0006508:proteolysis and peptidolysis	0.43

OAS1 †	ENSG00000089127:2',5'-OLIGOADENYLATE SYNTHETASE 1	GO:0006955:immune response	0.42

SERPINH1	ENSG00000149257:COLLAGEN-BINDING PROTEIN 2 PRECURSOR	GO:0006950:response to stress	0.39

CYBA	ENSG00000051523:CYTOCHROME B-245 ALPHA	GO:0006118:electron transport; GO:0006801: superoxide metabolism	0.38

IFITM2 †	ENSG00000185201:INTERFERON-INDUCED TRANSMEMBRANE PROTEIN 2	GO:0006955:immune response	0.34

IGFBP3	ENSG00000146674:INSULIN-LIKE GROWTH FACTOR BINDING PROTEIN 3 PRECURSOR	GO:0007165:signal transduction; GO:0001558:regulation of cell growth	0.31

ALDH1A1	ENSG00000165092:ALDEHYDE DEHYDROGENASE 1 FAMILY MEMBER A1	GO:0006081:aldehyde metabolism	0.29

IFITM1 †	ENSG00000185885:INTERFERON-INDUCED TRANSMEMBRANE PROTEIN 1	GO:0006955:immune response; GO:0008285:negative regulation of cell proliferation	0.25

*G1P2 †	ENSG00000182106:UBIQUITIN CROSS-REACTIVE PRECURSOR	GO:0006955:immune response; GO:0007267:cell-cell signaling	0.17

**Figure 6:**
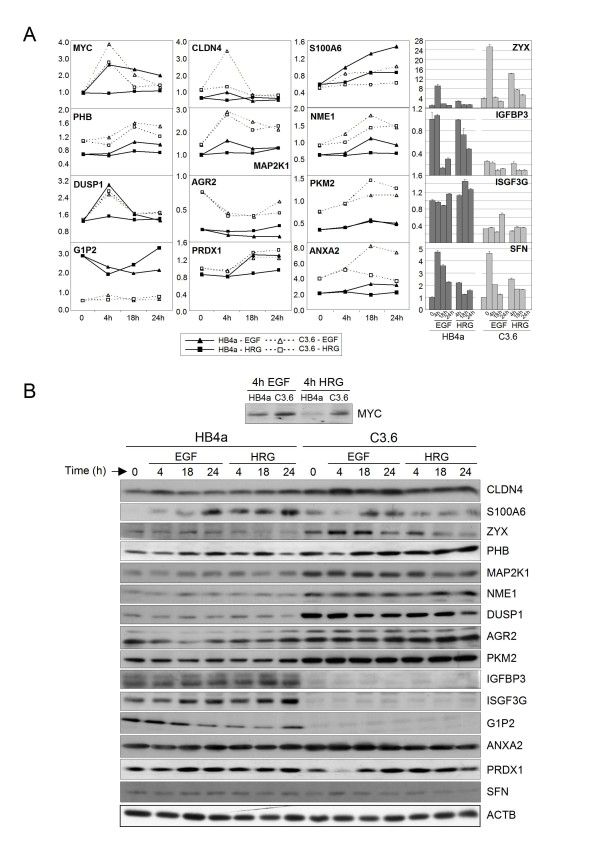
**Immunoblot validation of differentially expressed genes**. A. Relative gene expression for 16 selected genes by microarray or real time RT-PCR analysis. B. Protein expression for these gene products by immunoblotting. Representative blots from 3-5 independent experiments are shown, including a beta-actin loading control. Myc protein expression was only examined at the 4 hr timepoint and was not detected at other time-points. Relative quantification of immunoblotting data is shown in Additional file 6.

Gene symbol, name, function and fold-change in expression (C3.6 vs. HB4a) at T0 are shown for genes displaying differential expression between cell lines and which were relatively unresponsive to growth factor. *Indicates that the protein product of this gene displays the same directionality of differential expression (data from 2D-DIGE and immunoblotting experiments ([38,72,78] and Figure 6)). ^† ^Indicates the 8 down-regulated interferon-stimulated genes (ISGs).

### Validation of gene expression changes

A set of genes of interest from the microarray analysis were chosen for validation using semi-quantitative real-time PCR and/or immunoblotting. In general, there was good agreement between the microarray and RT-PCR datasets for genes examined, although fold-changes were generally higher and data more reproducible for the RT-PCR analyses (Fig. 5). Thus, EGF and/or HRG treatment induced AREG, VEGF, SFN, VIL2, ZYX and CTSC expression, with a more potent induction of VIL2 and ZYX in C3.6 cells, in agreement with the microarray data. Also in agreement, IGFBP3 and three ISGs (ISGF3G, G1P2 and OAS1) were all expressed at lower levels in C3.6 cells with IGFBP3 potently down-regulated by both GFs in the HB4a cells, whilst S100P was overexpressed in the C3.6 cells. One exception was vimentin (VIM), where RT-PCR showed increased expression in C3.6 cells with potent induction by EGF and HRG, rather than the down-regulation suggested from the microarray. The reason for this discrepancy is unclear, although the RT-PCR data is likely to provide a more accurate measure of regulated expression. The protein expression of several targets was also examined to test if the observed mRNA changes were indeed translated at the protein level (Fig. 6 and Additional file 6). When comparing the effect of ErbB2 overexpression alone (i.e. between cell lines), there was reasonable concordance between relative protein and mRNA expression for MYC, CLDN4, ZYX, PHB, MAP2K1, NME1, AGR2, PKM2 and ANXA2, (all up-regulated) and IGFBP3, ISGF3G and G1P2 (all down-regulated) (Fig. 6B). However, GF-induced changes in protein level were only apparent for MYC, CLDN4, S100A6, ZYX and G1P2 (Fig. 6C). In particular, the robust repression of IGFBP3 mRNA by GF treatment was not observed at the protein level or the induction of DUSP1 and SFN.

### Altered expression of IGFBP3 may alter IGF1-dependent signalling

**Figure 7:**
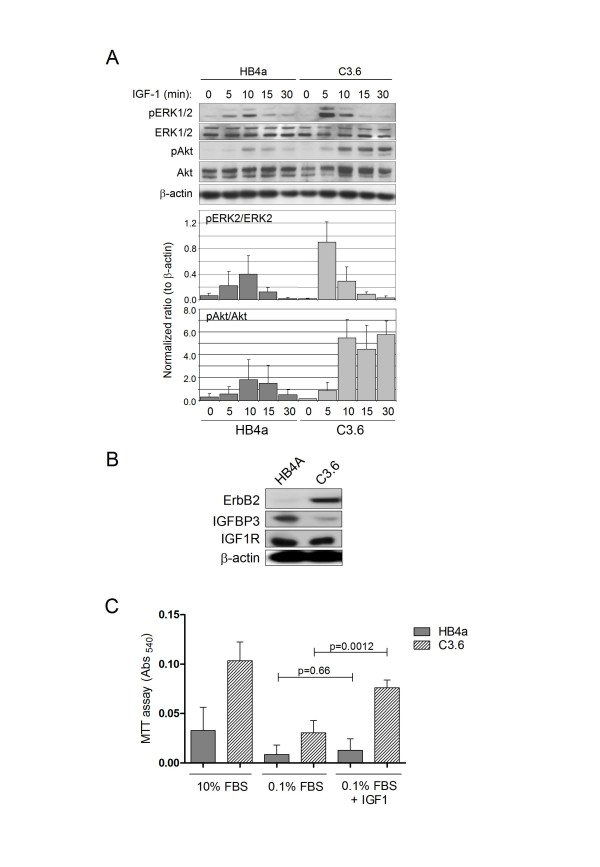
**ErbB2-enhances IGF1 signalling and proliferation in the HMLEC system**. A. Cells were starved of serum for 48 hrs and then stimulated with 25 ng/mL IGF1 for the indicated times. Activation of ERK1/2 and Akt was assessed by immunoblotting with phospho-specific antibodies and protein levels checked by re-probing membranes with non-phospho-specific and beta-actin antibodies. Blotting data was quantified by densitometry. Intensities for each band were normalized to the actin band in that lane and the ratios pAkt/Akt and pERK2/ERK2 calculated. Normalized ratios were then averaged from 3 independent blots and plotted using standard deviation as the error. B. Levels of ErbB2, IGFBP3 and IGF1R in HB4a and C3.6 cells were assessed by immunoblotting. C. MTT proliferation assays were carried out on HMLECs in media supplemented with 10% FBS, 0.1% FBS and 0.1% FBS plus 25 ng/mL IGF1 over a period of 48 hrs.

**Figure 8:**
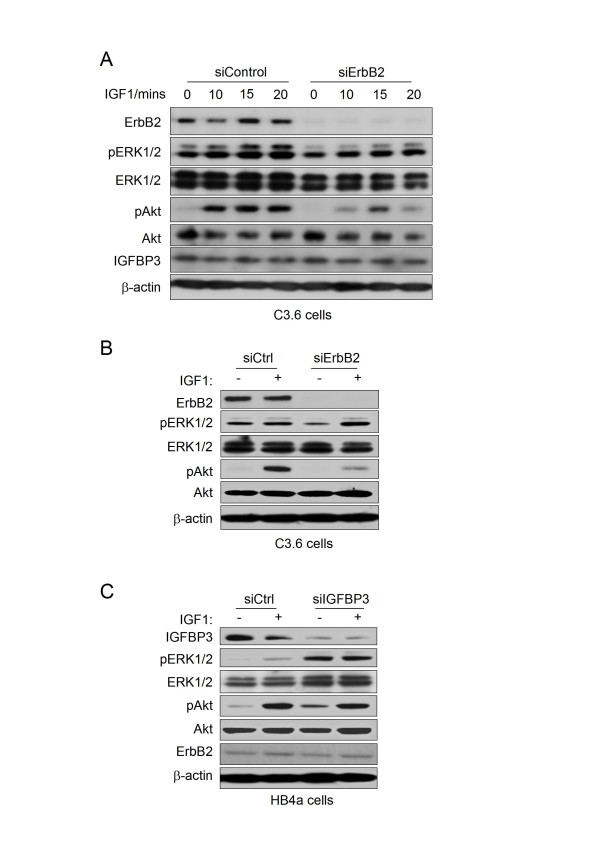
**Effect of siRNA-mediated knockdown of ErbB2 expression on IGF1-stimulated signalling**. A. Control siRNA and siErbB2-transfected C3.6 cells were serum starved for 48 hrs and then stimulated with 25 ng/mL IGF1 for the indicated times. Activation of ERK1/2 and Akt was assessed by immunoblotting with phospho-specific antibodies and protein levels checked by re-probing membranes with non-phospho-specific, ErbB2, IGFBP3 and beta-actin antibodies. B. Control siRNA and siErbB2-transfected C3.6 cells were serum starved for 48 hrs and then stimulated with 25 ng/mL IGF1 for 20 min (+) or left unstimulated (-). Lysates were immunoblotted as in A. C. Control siRNA and siIGFBP3-transfected HB4a cells were serum starved for 48 hrs and then stimulated with 25 ng/mL IGF1 for 20 min (+) or left unstimulated (-). Lysates were immunoblotted as in A.

Given the reduced expression of IGFBP3 in the C3.6 cells, IGFBP3's reported role in regulating IGF activity [35] and the reported aberrant regulation of the IGF system in breast cancer [36], we wanted to further examine if IGF1-induced signalling might be affected by ErbB2 overexpression in these cells. Cells were stimulated with IGF1 and proliferative and survival signalling assessed by immunoblotting with antibodies specific to the phosphorylated, activated forms of ERK1/2 and Akt. ERK2 phosphorylation was induced more rapidly in the C3.6 cells, though by 10 min the levels were similar in the two cell lines (Fig. 7A). Akt phosphorylation was also induced more rapidly, reaching a higher level and was sustained in the C3.6 cells. These effects were not due to altered expression of the IGF1 receptor (IGFR), which was equivalent in the two cell lines (Fig. 7B). These data show that early IGF1-dependent signalling events are enhanced in the ErbB2-overexpressing cells and may contribute to the enhanced proliferation we observed in the C3.6 cells in response to IGF1 treatment (Fig. 7C). We next assessed the involvement of ErbB2 in this phenomenon by targeting ErbB2 knockdown by transient reverse transfection with specific siRNAs and examined IGF1 signalling responses by immunoblotting (Fig. 8). Knockdown of ErbB2 expression in C3.6 cells (>90%) resulted in a modest reduction of both basal and IGF1-induced ERK1/2 phosphorylation and substantial reduction of IGF1-induced Akt phosphorylation (Fig. 8A and 8B). There was no effect of ErbB2 knockdown or IGF1 treatment on intracellular IGFBP3 levels. Notably, partial knockdown of IGFBP3 (~60%) in the HB4a cells resulted in both enhanced basal ERK1/2 and Akt phosphorylation, with little effect on IGF1-stimulated levels (Fig. 8C). Knockdown of IGFBP3 in C3.6 had little effect on IGF1-dependent signalling, presumably as its levels are already very low in these cells (data not shown). Taken together, these data reveal a positive role for ErbB2 expression in enhancing IGF1-dependent signalling in these HMLECs, and also suggested that IGFBP3 acts a negative regulator of this signalling.

**Figure 9:**
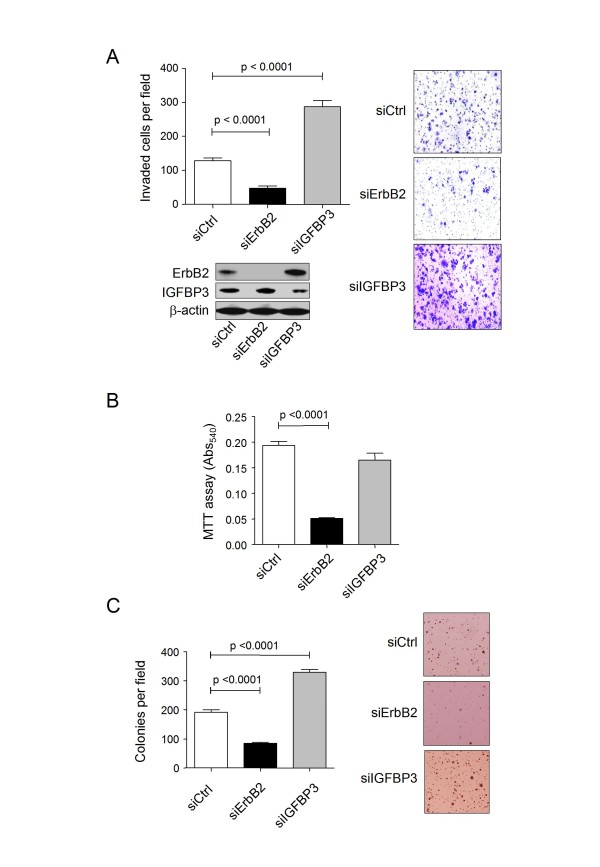
**Effect of siRNA-mediated knockdown of ErbB2 and IGFBP3 expression on invasiveness, proliferation and anchorage independent colony formation in SKBR3 cells**. A. Control siRNA, siErbB2- and siIGFBP3-transfected SKBR3 cells were subjected to a Matrigel-based invasion assay as described in the Methods section. The graph shows the number of invaded cells per field for each condition. Images of stained invaded cells are shown on the right. Knockdowns were confirmed by immunoblotting. B. An MTT-based proliferation assay was carried out on control siRNA, siErbB2- and siIGFBP3-transfected SKBR3 cells in complete media over 48 hrs. C. Control siRNA, siErbB2- and siIGFBP3-transfected SKBR3 cells were assayed for anchorage-independent growth using a soft agar colony forming assay (see Methods section). The graph shows the average number of colonies per field, whilst the images show representative microscopy fields.

To further address the functional consequences of a possible link between ErbB2 and IGFBP3 regulation and a possible role in breast cancer, cell-based assays were performed in the ErbB2-overexpressing and invasive breast cancer cell line SKBR3 after knocking down ErbB2 and IGFBP3 expression. Knockdown of ErbB2 expression (>95%) resulted in reduced cell invasion through matrigel (Fig. 9A), reduced proliferation (Fig. 9B) and reduced anchorage-independent colony formation (Fig. 9C), confirming the dependence of this cell line on ErbB2 overexpression for its transformed phenotype. Conversely, siRNA-mediated knockdown of IGFBP3 in these cells (~50%), resulted in increased (~2.3-fold) invasion (Fig. 9A) and increased (~1.75-fold) anchorage-independent colony formation (Fig. 9C), whilst proliferation in normal media was not significantly altered (Fig. 9B). We conclude that IGFBP3 is an inhibitor of tumourigenic phenotype and that ErbB2 may promote transformation, at least in part, through suppression of IGFBP3 expression and promotion of IGF1-dependent signalling.

## Discussion

This study has identified genes whose differential expression may contribute to ErbB2-dependent transformation and which define common and specific signalling events induced through EGFR and ErbB3 receptor-containing complexes. Although we and others have previously examined ErbB2-dependent gene expression changes in the same cell model, and find overlap in the genes identified [37,38], to the best of our knowledge, this is the first study to simultaneously investigate long-term ErbB2- and GF-dependent gene expression using ligands that activate specific ErbB receptor complexes in the same cell system. A number of gene expression changes were further validated using qRT-PCR and we report a good correlation between the datasets, indicating the robustness of the microarray protocol employed.

There were significantly more HRG-responsive genes than EGF-responsive genes and in many cases the HRG response was elevated in the ErbB2-overexpressing cells. This is likely to be a consequence of the higher expression of ErbB2 and ErbB3 in these cells [32] and the preferred heterodimerzation of these receptors [3-6], which would act to augment the response to HRG. We do not think that ErbB4 (also a HRGβ1 receptor) plays a major role in orchestrating signalling events in this cell system, since it appears to be expressed at very low levels, if at all, in these cell lines (data not shown). Although HRG-induced expression was generally of a lower magnitude than for EGF, it was often sustained compared to EGF, consistent with our previous finding that HRG-dependent mitogenic signalling is sustained in these cells [32]. Such temporal differences may be connected with differential rates of receptor or signal down-regulation, but also highlight the fact that the two growth factors initiate diverse responses which are likely to be relevant *in vivo*. Genes induced robustly by HRG (ZNF236, ZFP36L1, ZFP36L2, MADH4, TRIO, HMGCR, SLC16A1, SLPI, GYS1, SFRS5, CTNND1, LCAT, LYN, STAT1, KRT15, C20orf16 and FN1) are likely candidates for regulation by the PI3K/Akt pathway which is potently activated by HRG through ErbB2-ErbB3 heterodimers [7,8]. Since HRG expression itself correlates with tumourigenicity and metastasis in breast cancer cells lines [39,40], it will be interesting to assess whether the induction of these genes is affected by chemical inhibition of the PI3K pathway, or whether such inhibitors would make clinically useful therapeutics for breast cancer treatment.

Notably, a group of EGF-specific genes (e.g. AREG, S100A2 and CTSC) were induced exclusively in the HB4a cells, potentially through EGFR homodimers which predominate in these cells [32]. One of these genes, AREG, is a ligand of EGFR itself, suggesting that EGF could drive autocrine signalling to enhance EGF-specific responses. Members of the MT family were also potently induced by EGF. Whilst induction of MT1 expression by EGF has been shown in rat hepatocytes [41], this is the first report of MT1 (and MT3) induction by EGF in human epithelial cells. Since the altered expression of MT family members has been implicated in neoplasia and drug resistance [42,43], it will be interesting to investigate whether MT expression is linked to deregulated GF signalling in cancer.

Many of the identified genes have been previously implicated in tumour progression, found to be aberrantly expressed in different tumour types and/or to be linked with poor prognosis, hyper-proliferation, cell survival or tumour invasiveness. Our findings suggest that dysregulated ErbB signalling can account for changes in the expression of these genes, and may thus contribute to the establishment and progression of ErbB2-overexpressing breast tumours. For example, of the genes induced by both GFs and augmented by ErbB2, the proto-oncogenic transcription factor MYC has been associated with many forms of cancer often indicating poor prognosis [44]. Importantly, patient survival was significantly reduced in breast cancers where MYC and ErbB2 are co-amplified [45]. The MYC-induced glycoprotein EMP1 was also similarly regulated and whilst its function is unknown, it has reported tumourigenic activity [46] and was identified as a marker of gefinitib-resistance in xenograft models [47]. Thus, one possible scenario that warrants further investigation is that EMP1 acts in concert with MYC to promote ErbB2-dependent proliferation and drug resistance. A pattern of ErbB2-augmented GF-induction was also observed for other genes known to be involved in proliferation, autocrine signalling and anti-apoptosis (e.g. ATF4, FOSL1, IER3, MAP2K1/MEK1, MAP2K3/MEK3, PDGF, TNFAIP3, VEGF) and it is possible that these changes contribute to the reported hyper-proliferative phenotype of these ErbB2-overexpressing cells [31,32]. Induction of the pro-angiogenic factor VEGF is particularly relevant to tumour progression and confirms previous data [48,49]. Notably, VEGF expression was shown to depend upon ATF4 expression under certain conditions [50] and we hypothesize that such a regulatory circuit exists in these cells, whereby ErbB2-augmented GF signalling would promote VEGF expression through up-regulation of ATF4. The induction of some genes was perhaps surprising given their reported functions. GADD45A, SFN and the dual-specificity phosphatases DUSP1/MKP1 and DUSP5 were induced by GF treatment and are involved in genotoxic stress-induced growth arrest [51], p53-dependent negative regulation of G2/M progression [52] and down-regulation of MAPK signalling, respectively [53]. We propose that these may be negative feedback mechanisms adapted to self-regulate proliferative signalling.

Conversely, the down-regulation of genes with anti-proliferative functions identifies mechanisms by which increased ErbB2 signalling may promote proliferation and survival. Examples include the multiple ISGs that were identified and IGFBP3. G1P2/ISG15 was the most down-regulated gene in the dataset. Like ubiquitin, G1P2 is conjugated to proteins in a process called ISGylation which appears to modulate protein activity during the immune response and signalling [54]. The other ISGs were UBE2L6 (the proposed E2 enzyme for ISGylation [55]), IFIT1, IFITM1, IFITM2, OAS1 and ISGF3G/p48/IRF9. Notably, ISGF3G is a component of a transcription factor complex that with STAT1 and STAT2 controls type I IFN-mediated induction of ISGs containing interferon-stimulated regulatory elements (ISREs) [56]. The lowered expression of ISGF3G could thus account for the down-regulation of the other ISGs in the ErbB2-overexpressing cells, as suggested by our previous work [38]. Whilst the ISGs were induced by IFN treatment in the HMLECs, induction of ISGF3G (particularly with IFNγ) was blocked by GF co-treatment, revealing a possible cross-talk between the IFN and ErbB signalling pathways (data not shown). Although preliminary, our data suggested an inverse correlation between ErbB2 and ISG expression, supporting a role for repressed basal ISG expression in the pathogenesis of ErbB2-dependent breast cancer.

IGFBP3 mRNA and protein expression were both markedly lower in the ErbB2-overexpressing cells, whilst mRNA levels were decreased by GF treatment, particularly in the parental cells. Given IGFBP3's putative role as a negative regulator of IGF1 signalling [35], its anti-proliferative role [57] and the negative correlation between serum IGFBP3 levels and cancer risk [58-60], we investigated a possible link between its expression and IGF1 signalling. We found that IGF1-mediated ERK and Akt activation and proliferation were increased in the ErbB2-overexpressing cells and that the signalling effect was reversed by siRNA-mediated knockdown of ErbB2. The mechanism by which this occurs is unclear, although does not involve altered IGF1R expression, and may be mediated through interaction between ErbB receptors and IGF1R as previously reported in other cell models [61-63]. ErbB2 may also down-regulate IGFBP3 expression to promote IGF1 signalling. We propose that ErbB2-dependent suppression of IGFBP3 expression is a long-term adaptive response and would be the reason why IGFBP3 protein levels were not affected by transient ErbB2 knockdown. We speculate that this may be due to IGFBP3 promoter methylation, as previously reported for other cancers [64,65]. In the C3.6 cells, IGFBP3 expression is suppressed, allowing maximal IGF1 signalling through ErbB2-IGF1R interaction [61-63]. Knocking down ErbB2 in these cells therefore does not affect IGFBP3 levels, but abrogates IFG1 signalling. In HB4a cells, IGF1 signalling is restricted by normal IGFBP3 expression with knockdown of IGFBP3 enhancing basal ERK1/2 and Akt activation, thus supporting its role as a negative regulator of proliferation and survival. Although reduced IGFBP3 expression did not affect acute IGF1 triggering, our data partly support findings in primary and immortalized human esophageal cells, where EGF-mediated down-regulation of IGFBP3 was shown to determine cellular response to IGF1 [66]. However, this effect may be mediated by the as yet unknown IGF1-independent actions of IGFBP3 (reviewed in [67,68])

The observed increases in invasiveness and anchorage-independent growth of ErbB2-overexpressing SKBR3 cells following knockdown of IGFBP3 supports a role for IGFBP3 as a negative regulator of cellular transformation in breast cancer and we propose that its down-regulation is a mechanism whereby ErbB2 promotes tumour cell growth through increased IGF1-dependent proliferation, survival and invasion. Indeed, a requirement for IGF1 in EGF-mediated cell cycle progression has been shown in primary murine mammary epithelial cells [69]. Whilst an attractive model, other studies report that IGFBP3 can potentiate EGF-stimulated proliferation in MCF10A cells [70] and that IGFBP3 expression is associated with growth stimulation of T47D human breast cancer cells [71]. These differences may be explained by cell type-specific effects and are possibly dependent upon the extent of interaction with the ErbB receptor system [71]. Future experiments should explore the effects of overexpressing IGFBP3 on IGF1 signalling, proliferation, survival and invasion and to investigate the level of IGFBP3 promoter methylation in this cell system.

We have previously reported a high correlation between mRNA and protein expression for a subset of genes in these cell lines [38], and a previous proteomic study found reduced expression of GSTP1, PRDX5 and USP14 and increased expression of KRT13, ALDH1A3 and NME1 in the C3.6 cells [72], in agreement with the mRNA data presented here. In the present study, the mRNA expression of several targets (MYC, CLDN4, S100A6, ZYX, PHB, MAP2K1, NME1, AGR2, PKM2, IGFBP3, ISGF3G, G1P2 and ANXA2) correlated with altered protein expression, signifying that these changes are likely to be functionally relevant. However, correlation between protein and mRNA expression was not apparent for some targets in response to the GF treatments. For example, the repression of IGFBP3 mRNA by GF treatment was not confirmed at the protein level and neither was induction of DUSP1 or SFN. This suggests that the IGFBP3 protein may be relatively stable over the time course of the assay or that the DUSP1 and SFN mRNAs are not translated. Such post-transcriptional regulatory mechanisms are likely to be important, and whilst some mRNA changes appear to be redundant, they may be relevant in other circumstances, for example, during development, differentiation or stress.

A relatively large group of genes involved in regulating the cytoskeleton, cell adhesion and motility were identified. Whilst various patterns of gene expression were apparent, genes up-regulated to a greater degree by either GF in the ErbB2-overexpressing cells (ZYX, VIM, VCL, TAGLN, VIL2, PDLIM1, ITGA2, ITGA3, PLAT, PLAUR, SERPINE1 and ANXA2) are perhaps the most interesting, since they may promote the ErbB2-mediated anchorage-independent growth and reduced cellular adhesion previously observed in this cell model system [31,38]. Notably, some of these genes are members of the plasminogen activator system and have been implicated in tumour progression and invasiveness through proteolysis of the extracellular matrix. Indeed, increased levels of PLAUR and SERPINE1 have been associated with poor prognosis in breast cancer patients [73,74]. Our data thus implicates ErbB2-mediated signalling in the regulation of the plasminogen activator system, as well as cell adhesion-related events.

Finally, a number of genes of unknown or poorly-defined function were identified and several were validated. These include BCAR3, CPNE3, CSRP1, HPCAL1, LCP1, MGC10471, NME1, SMAP, ZFP36L1, ZFP36L2 and ZNF236, which were differentially regulated by GF in an ErbB2-dependent manner and AGR2, LOC402057, NPC2, PSCA, S100P and SERF2, which were differentially expressed in an ErbB2-dependent manner. Our data reveals that the expression of these genes can be regulated by ErbB receptor signalling and thus implicates them as possible biomarkers and effectors of ErbB2-dependent tumourigenesis. Indeed, AGR2, LCP1 and S100P overexpression have been previously correlated with breast cancer progression [75-77], and we now link the aberrant expression of these genes with ErbB2 expression.

## Conclusions

Fully understanding and characterising the interactions and outcomes of the identified gene expression changes is a huge undertaking and will require additional studies addressing the functional consequences of such changes. However our data provides a valuable resource and a number of testable hypotheses with potentially important implications in GF signalling and ErbB2-dependent tumourigenesis. One assumption of this work is that the measured effects are indeed ErbB2-dependent and not an artefact of clonal selection and variation. With this in mind, future validation work should involve testing of candidate genes in other clones, mammary cell lines or breast tumour samples that overexpress ErbB2 and by RNAi-mediated knockdown of ErbB2 expression to see if the observed effects can be reversed. Indeed, for one candidate, IGFBP3, we demonstrate it to be a negative regulator of transformation using siRNA-dependent knockdown and propose that its down-regulation enhances IGF1-dependent signalling in ErbB2-overexpressing cells.

## Abbreviations

GF: growth factor; HRG: heregulinβ1; HMLEC: human mammary luminal epithelial cell; FCS: foetal calf serum, qRT-PCR: quantitative real time-PCR; siRNA: small interfering RNA; MT: metallothionein; IGF1: insulin-like growth factor 1; ISG: interferon-stimulated gene; IFN: interferon; IGFBP3: Insulin-like growth factor binding protein 3

## Declaration of Competing interests

The authors declare that they have no competing interests.

## Authors' contributions

JW carried out the molecular functional analysis of IGFBP3 and IGF1 signalling and helped to draft the manuscript. MB carried out the microarray experiments and conducted the statistical analysis, qRT-PCR and some of the protein validation work. H-LC conducted preliminary IGF1 signalling work and protein validation, BG participated in microarray data analysis by constructing the Excel macro for visualisation of data and data mining. JT conceived of the study, participated in its design and coordination and also drafted the manuscript. All authors read and approved the final manuscript.

## Pre-publication history

The pre-publication history for this paper can be accessed here:

http://www.biomedcentral.com/1471-2407/10/490/prepub

## Supplementary Material

Additional file 1 **List of antibodies, sources and working dilutions used for immunoblotting**. This Word DOC displays a list of antibodies, sources and working dilutions used for immunoblotting. Click here for file

Additional file 2 **Full gene lists (775 genes from SAM) grouped by responsiveness to EGF and HRG**. This list has hyperlinks to the SOURCE gene database and an excel macro is available upon request for graphical visualization of expression. Click here for file

Additional file 3 ***K*-means and hierarchical clustering of EGF and HRG-responsive genes**. *K*-means clustering was performed as described in Figure 4, with all 4 groups of genes subjected to hierarchical clustering. Click here for file

Additional file 4 ***K*-means and hierarchical clustering of EGF responsive genes**. *K*-means clustering was performed as described in Figure 4 using only the EGF-responsive genes generated by SAM. Groups (iii) and (iv) were then subjected to hierarchical clustering. Click here for file

Additional file 5 ***K*-means and hierarchical clustering of HRG responsive genes**. *K*-means clustering was performed as described in Figure 4 using only the HRG-responsive genes generated by SAM. Groups (i) and (iv) were then subjected to hierarchical clustering. Click here for file

Additional file 6 **Relative quantification of immunoblotting data**. All membranes were re-probed for beta-actin and densitometry performed on all bands using local background subtraction. Intensities for each band were normalized to the actin band in that lane and normalized values were averaged from 3-5 independent blots and plotted using the standard deviation as the error. Click here for file
