# Supplementary material for: Transcriptional profiling of ErbB signalling in mammary luminal epithelial cells - interplay of ErbB and IGF1 signalling through IGFBP3 regulation
Source: BMC Cancer. 2010 Sep 14;10:490. doi: 10.1186/1471-2407-10-490 (PMC2946312; doi:10.1186/1471-2407-10-490)
Supplement: Additional file 1 — List of antibodies, sources and working dilutions used for immunoblotting. This Word DOC displays a list of antibodies, sources and working dilutions used for immunoblotting. [file 1471-2407-10-490-S1.DOC]

| **Antibody** | **MW** | **Company/Source** | **Antibody species** | **Dilution for WB** |
| --- | --- | --- | --- | --- |
| ACTB/Actin beta (AC-15) | 42 | Sigma | mo mAb | 1:25000 |
| AGR2 | 22 | Abnova | mo mAb | 1:1000 |
| AKT | 60 | Cell Signaling Technology | rab pAb | 1:2000 |
| AKT active (pSer473) | 60 | Cell Signaling Technology | rab pAb | 1:2000 |
| ANXA2/Annexin II | 36 | Transduction Laboratories | mo mAb | 1:5000 |
| CLDN4/Claudin 4 (C-18) | 23 | Santa Cruz | goat pAb | 1:1000 |
| DUSP1/MKP1 (C-19) | 40 | Santa Cruz | rab pAb | 1:1000 |
| ErbB2/Neu (C-18) | 185 | Santa Cruz | rab pAb | 1:500 |
| ERK1/2 | 42/44 | Promega | rab pAb | 1:5000 |
| ERK1/2 activate (pT183/pY185-ERK2) | 42/44 | Promega | rab pAb | 1:2000 |
| G1P2/ISG15 | 15 | Santa Cruz | mo mAb | 1:1000 |
| IGF1Rβ (C-20) | 97 | Santa Cruz | rab pAb | 1:1000 |
| IGFBP3 (H-98) | 35 | Santa Cruz | rab pAb | 1:200 |
| ISGF3γ/p48) | 48 | Santa Cruz | rab pAb | 1:1000 |
| MAP2K1/MEK1 | 45 | Cell Signaling Technology | rab pAb | 1:1000 |
| Myc (clone 9E10) | 55 | Santa Cruz and CRUK | mo mAb | 1:500 |
| NME1/NM23-H1 (C-20) | 23 | Santa Cruz | rab pAb | 1:1000 |
| PHB/Prohibitin (H80) | 30 | Santa Cruz | rab pAb | 1:1000 |
| PKM2/M2-PK (DF4) | 57 | ScheBo Biotech | mo mAb | 1:1000 |
| PRDX1/Peroxiredoxin 1 | 23 | Lab Frontier | rab pAb | 1:2000 |
| S100A6 (calcyclin) | 10 | Sigma | mo mAb | 1:500 |
| SFN/stratifin/14-3-3 sigma (C-18) | 28 | Santa Cruz | goat pAb | 1:1000 |
| STAT1 (E-23) | 84/91 | Santa Cruz | rab pAb | 1:3000 |
| STAT1 active (pTyr701) | 91 | Cell Signaling Technology | rab pAb | 1:5000 |
| STAT3 | 89 | Cell Signaling Technology | rab pAb | 1:1000 |
| STAT3 active (pTyr705) | 89 | Cell Signaling Technology | rab pAb | 1:2000 |
| ZYX/Zyxin (C-19) | 85 | Santa Cruz | goat pAb | 1:1000 |
