# Supplementary material for: Transcriptional profiling of ErbB signalling in mammary luminal epithelial cells - interplay of ErbB and IGF1 signalling through IGFBP3 regulation
Source: BMC Cancer. 2010 Sep 14;10:490. doi: 10.1186/1471-2407-10-490 (PMC2946312; doi:10.1186/1471-2407-10-490)
Supplement: Additional file 3 — K-means and hierarchical clustering of EGF and HRG-responsive genes. K-means clustering was performed as described in Figure 4, with all 4 groups of genes subjected to hierarchical clustering. [file 1471-2407-10-490-S3.PPT]

## Slide 1
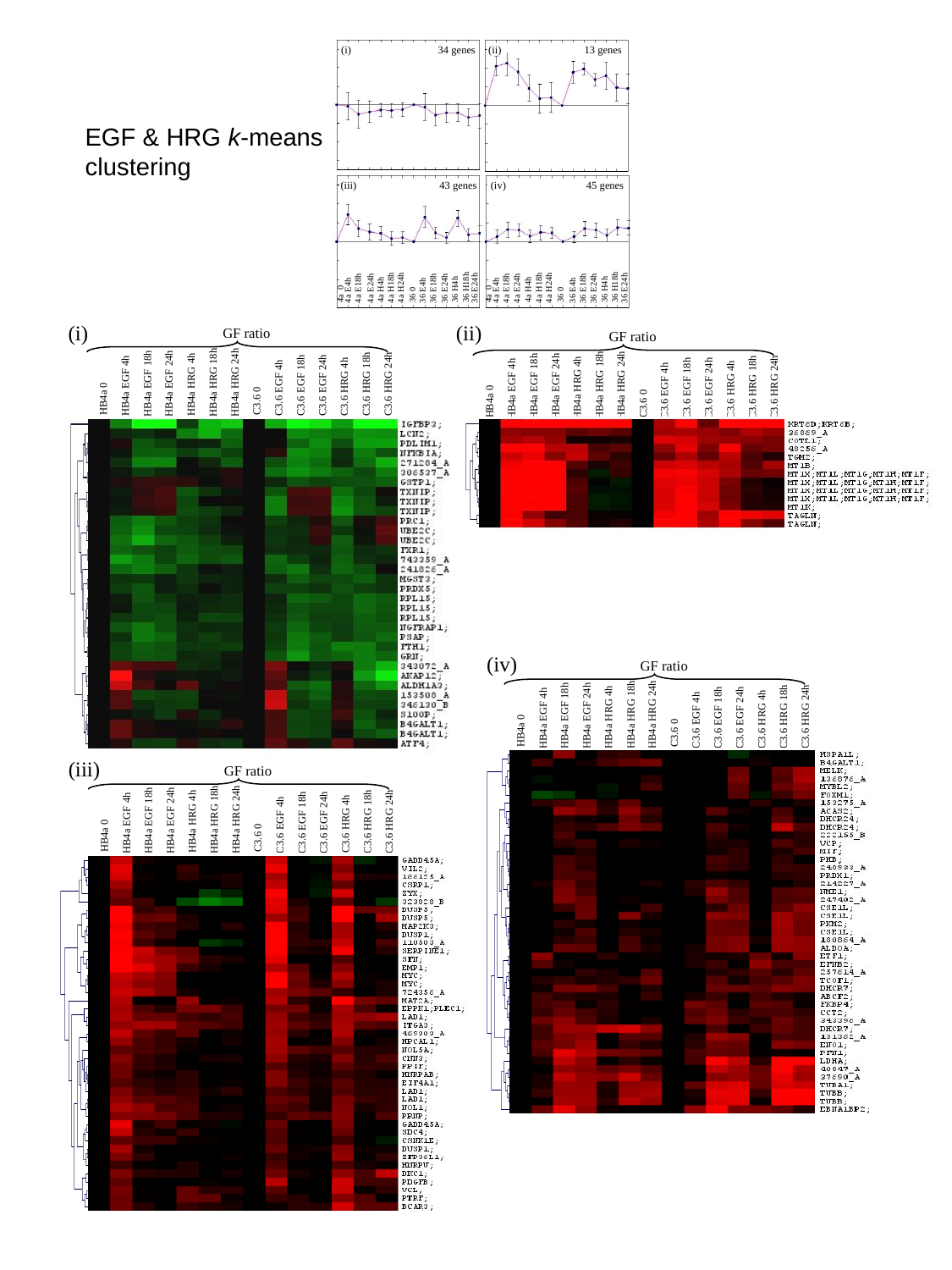

(i)
(ii)
34 genes
13 genes
(iii)
43 genes
(iv)
45 genes
36 H18h
36 H18h
4a H18h
4a H24h
4a H18h
4a H24h
36 E18h
36 E24h
36 E24h
36 E18h
36 E24h
36 E24h
4a E18h
4a E24h
4a E18h
4a E24h
36 H4h
36 H4h
4a H4h
4a H4h
36 E4h
36 E4h
4a E4h
4a E4h
4a 0
4a 0
36 0
36 0
EGF & HRG k-means
clustering
(i)
(ii)
GF ratio
HB4a HRG 18h
HB4a HRG 24h
HB4a EGF 18h
HB4a EGF 24h
C3.6 HRG 18h
C3.6 HRG 24h
HB4a HRG 4h
C3.6 EGF 18h
C3.6 EGF 24h
HB4a EGF 4h
C3.6 HRG 4h
C3.6 EGF 4h
HB4a 0
C3.6 0
GF ratio
HB4a HRG 18h
HB4a HRG 24h
HB4a EGF 18h
HB4a EGF 24h
C3.6 HRG 18h
C3.6 HRG 24h
HB4a HRG 4h
C3.6 EGF 18h
C3.6 EGF 24h
HB4a EGF 4h
C3.6 HRG 4h
C3.6 EGF 4h
HB4a 0
C3.6 0
(iv)
GF ratio
HB4a HRG 18h
HB4a HRG 24h
HB4a EGF 18h
HB4a EGF 24h
C3.6 HRG 18h
C3.6 HRG 24h
HB4a HRG 4h
C3.6 EGF 18h
C3.6 EGF 24h
HB4a EGF 4h
C3.6 HRG 4h
C3.6 EGF 4h
HB4a 0
C3.6 0
(iii)
GF ratio
HB4a HRG 18h
HB4a HRG 24h
HB4a EGF 18h
HB4a EGF 24h
C3.6 HRG 18h
C3.6 HRG 24h
HB4a HRG 4h
C3.6 EGF 18h
C3.6 EGF 24h
HB4a EGF 4h
C3.6 HRG 4h
C3.6 EGF 4h
HB4a 0
C3.6 0
