# Supplementary material for: Transcriptional profiling of ErbB signalling in mammary luminal epithelial cells - interplay of ErbB and IGF1 signalling through IGFBP3 regulation
Source: BMC Cancer. 2010 Sep 14;10:490. doi: 10.1186/1471-2407-10-490 (PMC2946312; doi:10.1186/1471-2407-10-490)
Supplement: Additional file 4 — K-means and hierarchical clustering of EGF responsive genes. K-means clustering was performed as described in Figure 4 using only the EGF-responsive genes generated by SAM. Groups (iii) and (iv) were then subjected to hierarchical clustering. [file 1471-2407-10-490-S4.PPT]

## Slide 1
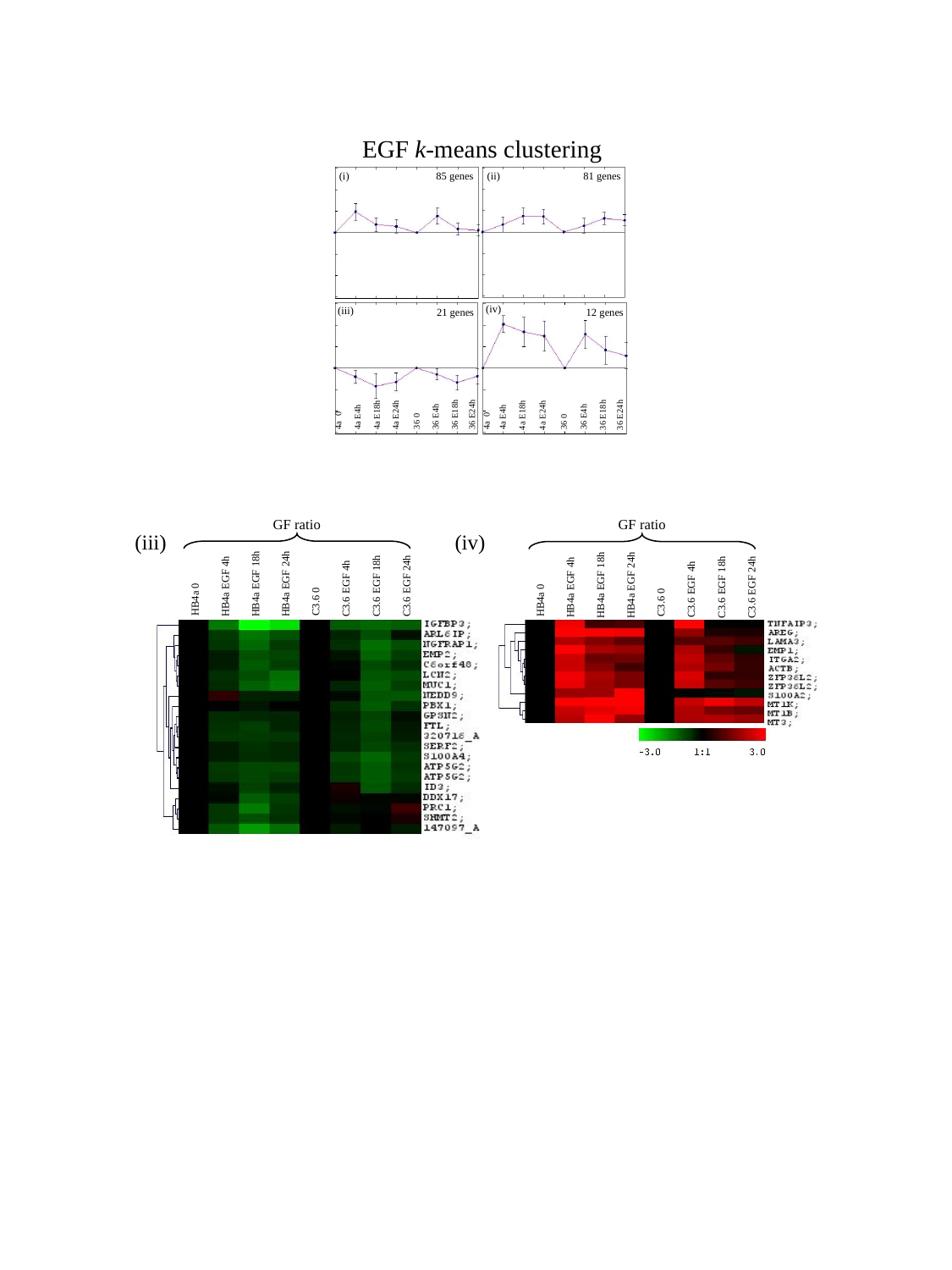

EGF k-means clustering
(i)
(ii)
85 genes
81 genes
(iv)
(iii)
21 genes
12 genes
36 E18h
36 E24h
36 E18h
36 E24h
4a E18h
4a E24h
4a E18h
4a E24h
36 E4h
36 E4h
4a E4h
4a E4h
4a 0
4a 0
36 0
36 0
GF ratio
HB4a EGF 18h
HB4a EGF 24h
C3.6 EGF 18h
C3.6 EGF 24h
HB4a EGF 4h
C3.6 EGF 4h
HB4a 0
C3.6 0
GF ratio
HB4a EGF 18h
HB4a EGF 24h
C3.6 EGF 18h
C3.6 EGF 24h
HB4a EGF 4h
C3.6 EGF 4h
HB4a 0
C3.6 0
(iii)
(iv)
