# Supplementary material for: Transcriptional profiling of ErbB signalling in mammary luminal epithelial cells - interplay of ErbB and IGF1 signalling through IGFBP3 regulation
Source: BMC Cancer. 2010 Sep 14;10:490. doi: 10.1186/1471-2407-10-490 (PMC2946312; doi:10.1186/1471-2407-10-490)
Supplement: Additional file 5 — K-means and hierarchical clustering of HRG responsive genes. K-means clustering was performed as described in Figure 4 using only the HRG-responsive genes generated by SAM. Groups (i) and (iv) were then subjected to hierarchical clustering. [file 1471-2407-10-490-S5.PPT]

## Slide 1
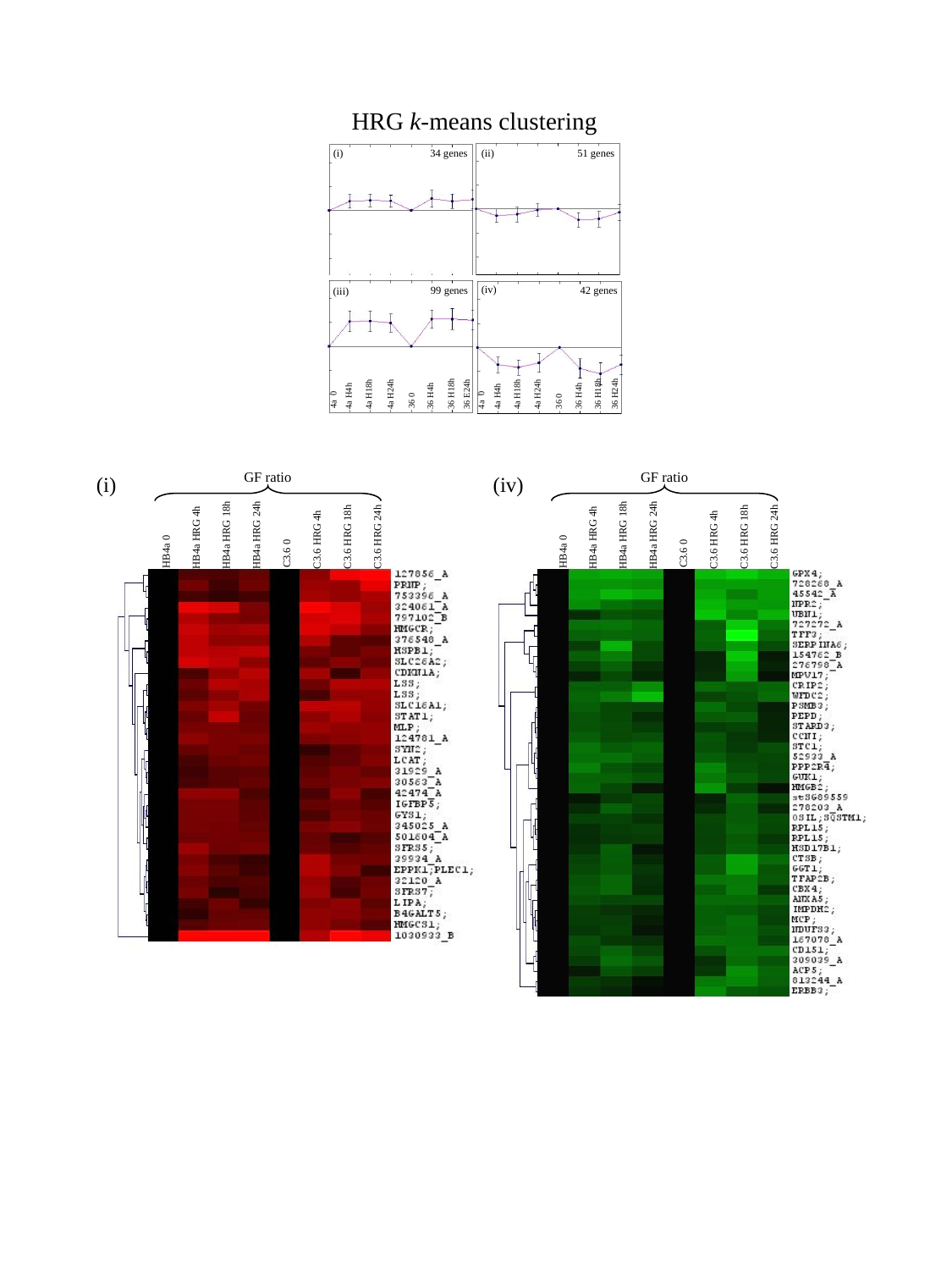

HRG k-means clustering
(i)
(ii)
34 genes
51 genes
(iv)
99 genes
42 genes
(iii)
36 H18h
36 H18h
36 H24h
4a H18h
4a H24h
4a H18h
4a H24h
36 E24h
36 H4h
36 H4h
4a H4h
4a H4h
4a 0
4a 0
36 0
36 0
GF ratio
HB4a HRG 18h
HB4a HRG 24h
C3.6 HRG 18h
C3.6 HRG 24h
HB4a HRG 4h
C3.6 HRG 4h
HB4a 0
C3.6 0
GF ratio
HB4a HRG 18h
HB4a HRG 24h
C3.6 HRG 18h
C3.6 HRG 24h
HB4a HRG 4h
C3.6 HRG 4h
HB4a 0
C3.6 0
(i)
(iv)
