# Supplementary material for: Transcriptional profiling of ErbB signalling in mammary luminal epithelial cells - interplay of ErbB and IGF1 signalling through IGFBP3 regulation
Source: BMC Cancer. 2010 Sep 14;10:490. doi: 10.1186/1471-2407-10-490 (PMC2946312; doi:10.1186/1471-2407-10-490)
Supplement: Additional file 6 — Relative quantification of immunoblotting data. All membranes were re-probed for beta-actin and densitometry performed on all bands using local background subtraction. Intensities for each band were normalized to the actin band in that lane and normalized values were averaged from 3-5 independent blots and plotted using the standard deviation as the error. [file 1471-2407-10-490-S6.PPT]

## Slide 1
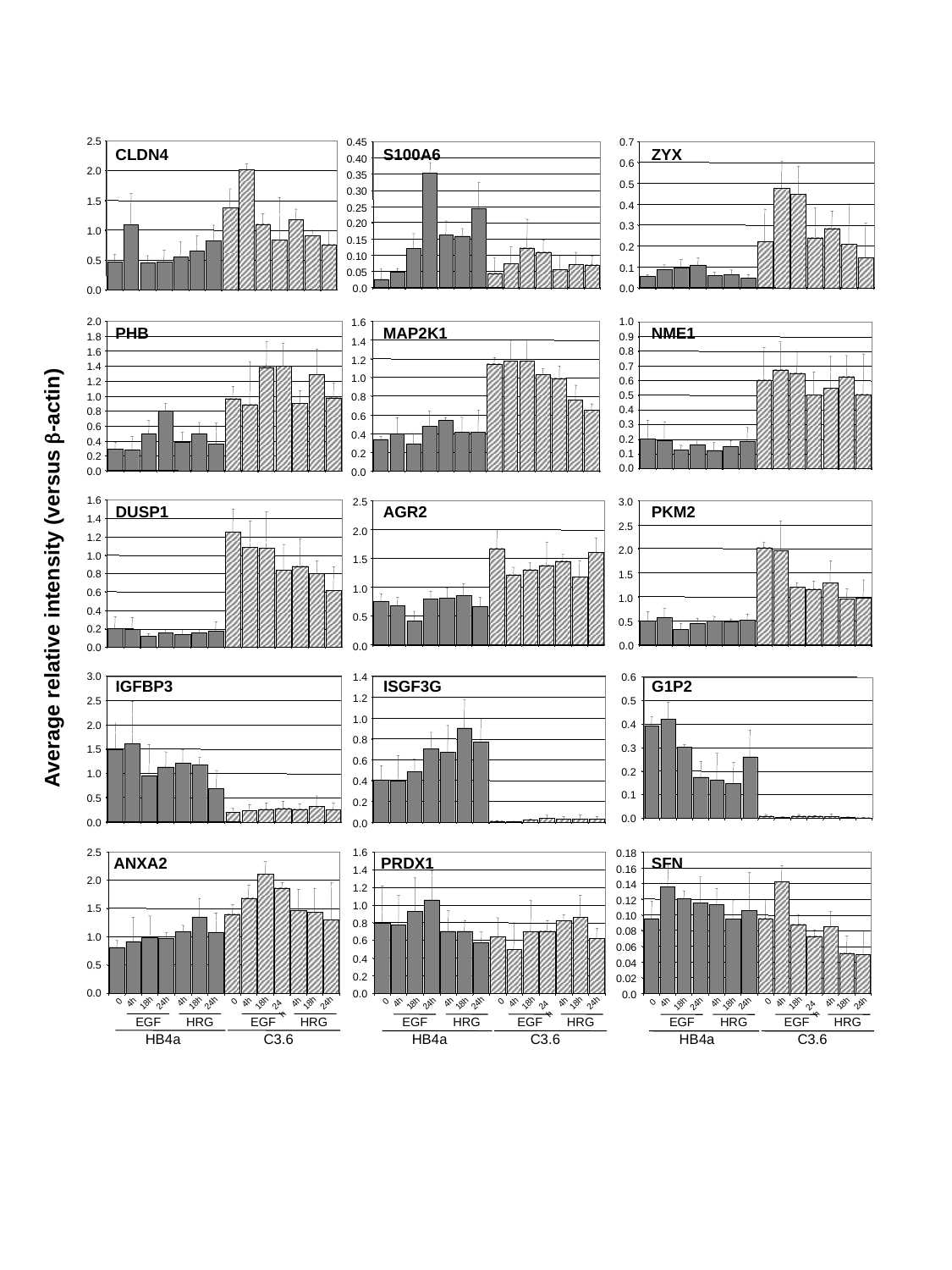

2.5
CLDN4
2.0
1.5
1.0
0.5
0.0
0.45
S100A6
0.40
0.35
0.30
0.25
0.20
0.15
0.10
0.05
0.0
0.7
ZYX
0.6
0.5
0.4
0.3
0.2
0.1
0.0
2.0
PHB
1.8
1.6
1.4
1.2
1.0
0.8
0.6
0.4
0.2
0.0
1.0
NME1
0.9
0.8
0.7
0.6
0.5
0.4
0.3
0.2
0.1
0.0
1.6
MAP2K1
1.4
1.2
1.0
0.8
0.6
0.4
0.2
0.0
1.6
DUSP1
1.4
1.2
1.0
0.8
0.6
0.4
0.2
0.0
2.5
AGR2
2.0
1.5
1.0
0.5
0.0
3.0
PKM2
2.5
2.0
1.5
1.0
0.5
0.0
Average relative intensity (versus β-actin)
3.0
IGFBP3
2.5
2.0
1.5
1.0
0.5
0.0
1.4
ISGF3G
1.2
1.0
0.8
0.6
0.4
0.2
0.0
0.6
G1P2
0.5
0.4
0.3
0.2
0.1
0.0
2.5
ANXA2
2.0
1.5
1.0
0.5
1.6
PRDX1
1.4
1.2
1.0
0.8
0.6
0.4
0.2
0.18
SFN
0.16
0.14
0.12
0.10
0.08
0.06
0.04
0.02
0.0
0.0
0.0
0
0
24h
4h
0
4h
4h
4h
0
24h
0
0
4h
24h
4h
4h
4h
4h
4h
4h
4h
18h
18h
24h
24h
18h
18h
24h
18h
18h
24h
24h
18h
18h
18h
24h
24h
18h
24h
18h
18h
24h
EGF
HRG
EGF
HRG
EGF
HRG
EGF
HRG
EGF
HRG
EGF
HRG
HB4a
C3.6
HB4a
C3.6
HB4a
C3.6
